# Young Sprague Dawley rats infected by *Plasmodium berghei*: A relevant experimental model to study cerebral malaria

**DOI:** 10.1371/journal.pone.0181300

**Published:** 2017-07-24

**Authors:** Sokhna Keita Alassane, Marie-Laure Nicolau-Travers, Sandie Menard, Olivier Andreoletti, Jean-Pierre Cambus, Noémie Gaudre, Myriam Wlodarczyk, Nicolas Blanchard, Antoine Berry, Sarah Abbes, David Colongo, Babacar Faye, Jean-Michel Augereau, Caroline Lacroux, Xavier Iriart, Françoise Benoit-Vical

**Affiliations:** 1 CNRS, LCC (Laboratoire de Chimie de Coordination), 205 route de Narbonne, Toulouse, France; 2 Université de Toulouse, UPS, INPT, Toulouse, France; 3 UFR Sciences de la Santé, Université Gaston Berger, St Louis, Sénégal; 4 CPTP (Centre de Physiopathologie de Toulouse Purpan), INSERM U1043, CNRS UMR5282, Université de Toulouse III, Toulouse, France; 5 UMR INRA ENVT 1225, Interactions Hôte Agent Pathogène, Ecole Nationale Vétérinaire de Toulouse, 23 Chemin des Capelles, Toulouse, France; 6 Laboratoire Hématologie, Centre Hospitalier Universitaire, Toulouse, France; 7 Service de Parasitologie-Mycologie, Centre Hospitalier Universitaire, Toulouse, France; 8 Hyphen-stat, 195 route d’Espagne, Toulouse, France; Instituto Oswaldo Cruz, BRAZIL

## Abstract

Cerebral malaria (CM) is the most severe manifestation of human malaria yet is still poorly understood. Mouse models have been developed to address the subject. However, their relevance to mimic human pathogenesis is largely debated. Here we study an alternative cerebral malaria model with an experimental *Plasmodium berghei* Keyberg 173 (K173) infection in Sprague Dawley rats. As in Human, not all infected subjects showed cerebral malaria, with 45% of the rats exhibiting Experimental Cerebral Malaria (ECM) symptoms while the majority (55%) of the remaining rats developed severe anemia and hyperparasitemia (NoECM). These results allow, within the same population, a comparison of the noxious effects of the infection between ECM and severe malaria without ECM. Among the ECM rats, 77.8% died between day 5 and day 12 post-infection, while the remaining rats were spontaneously cured of neurological signs within 24–48 hours. The clinical ECM signs observed were paresis quickly evolving to limb paralysis, global paralysis associated with respiratory distress, and coma. The red blood cell (RBC) count remained normal but a drastic decrease of platelet count and an increase of white blood cell numbers were noted. ECM rats also showed a decrease of glucose and total CO_2_ levels and an increase of creatinine levels compared to control rats or rats with no ECM. Assessment of the blood-brain barrier revealed loss of integrity, and interestingly histopathological analysis highlighted cyto-adherence and sequestration of infected RBCs in brain vessels from ECM rats only. Overall, this ECM rat model showed numerous clinical and histopathological features similar to Human CM and appears to be a promising model to achieve further understanding the CM pathophysiology in Humans and to evaluate the activity of specific antimalarial drugs in avoiding/limiting cerebral damages from malaria.

## Introduction

Malaria remains a “major killer of children” in one of its more severe aspects, Cerebral Malaria (CM), with a fatality rate of 15–25% in African children despite effective antimalarial chemotherapy [1]. CM causes 78% of all malaria deaths. It is caused by the apicomplexan parasite *Plasmodium falciparum*, affecting not only children under the age of 5, but also pregnant women and non-immune patients such as tourists in endemic areas [2–4]. This pathology is an acute encephalopathy characterized by fever, vomiting, headache, seizure, respiratory distress, malarial retinopathy, impaired consciousness and/or coma [2,5–7].

Despite its well-documented clinical signs, the pathogenesis of Human CM is still unclear. Moreover, human CM is not a single pathological entity. Establishing correlations between clinical signs and cerebral lesions requires autopsies that are not easily accepted in countries where CM is endemic, and consequently only rarely performed [8]. Therefore, a reliable surrogate is crucial to get better insights into the pathophysiology of Human CM. In this framework, non-human primates would be the best model for malaria research [9], but their use is limited by financial, technical and ethical constraints. Rodent models are widely used permitting brains studies at different phases of the illness.

Thanks to the mouse experimental model of *Plasmodium berghei* infection, some particular points of CM pathogenesis, such as brain tissue inflammation, were highlighted [10]. However, given the considerable differences between the mouse and Humans, especially concerning the immune response, the relevance of the malaria mouse model is largely debated [9–16]. Indeed, the different mouse strains used in the laboratory gave highly variable experimental infection responses. Moreover, the ECM histopathological signs in mice are different from those of humans, and more specifically with regard to parasite sequestration, which is considered a crucial issue in Human CM occurrence [13,17,18]. Furthermore, experimental mouse models are frequently condemned as poor predictors for translational studies especially for the development of experimental drugs that may be ineffective in human pathologies [16]. Hence, it would be useful to establish an accessible and more relevant ECM model than mouse.

In parasitic diseases such as schistosomiasis, the rat model showed strong immunological similarities to Humans [19]. Moreover, the rat has been described as a relevant model for malaria [20–24] and for a lot of neurological studies [25–27]. In this context we assessed the relevance of the rat model in the study of ECM pathophysiology. We infected Sprague Dawley (SD) rats by the *P*. *berghei* K173 (here written later K173) strain and analyzed the different features of the model such as neurological signs, breakdown of the blood brain barrier (BBB), histological brain damage, immune response and hematological parameters in relation with the parasitemia and features reported in Human CM cases.

## Results and discussion

### Clinical features of ECM in SD rat

**Fig 1:**
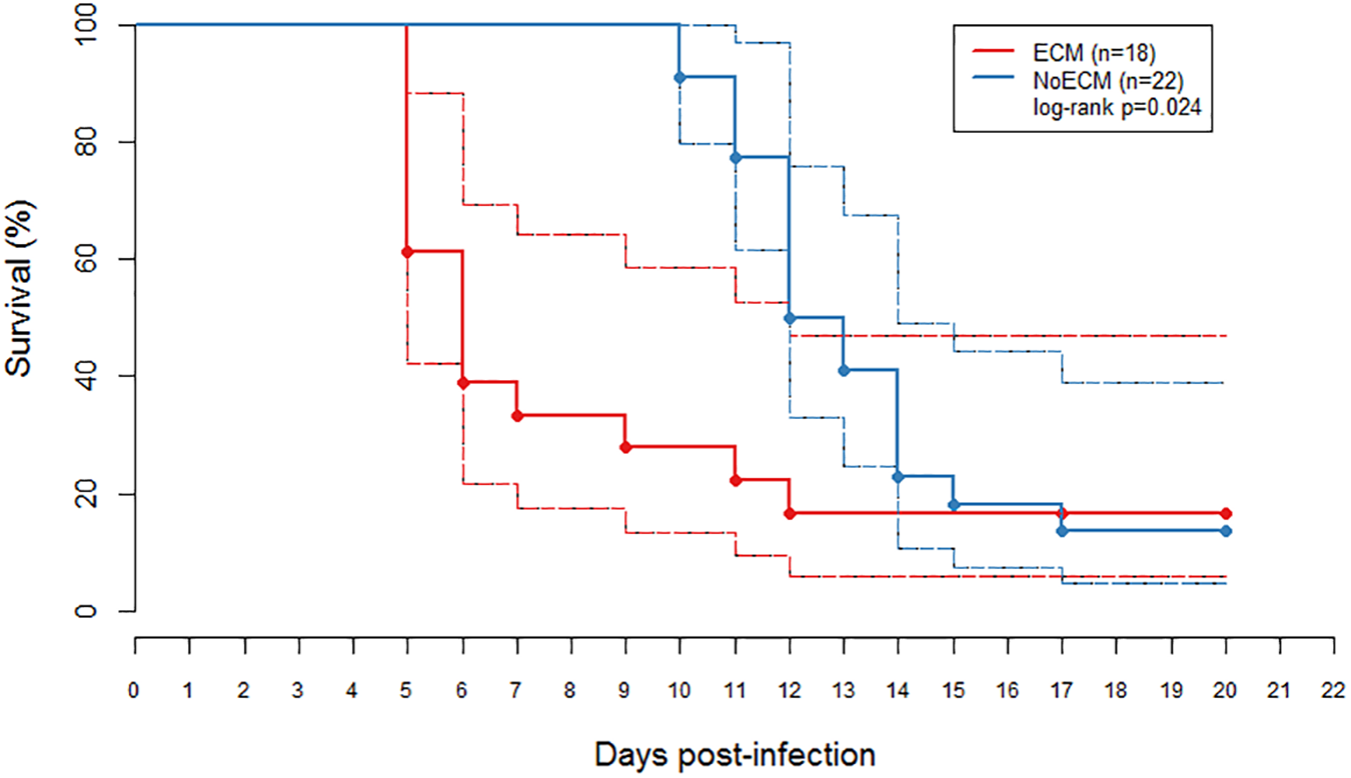
Kaplan-Meyer curve estimate of a survival, with 95% confidence limits, of young Sprague Dawley (SD) rats with Experimental Cerebral Malaria (ECM; n = 18) and without Experimental Cerebral Malaria (NoECM; n = 22) after K173 infection. The solid lines are the survival curves and the dashed lines are the confidence intervals. Except for 6 rats (3 ECM and 3 NoECM), which cured spontaneously and were sacrificed at day 20, all the infected rats died at the latest on the 17^th^ day *pi*. ECM rats died spontaneously between days 5 and 12 while the first cases of fatal outcomes for NoECM rats occurred on day 10. The difference between two groups was significant (p = 0.024).

Infected SD rats were monitored to characterize the clinical outcome of ECM on K173 infection. Globally, K173 infection led to a severe form of malaria and a fatal outcome for 85% of the SD rats (n = 34). Infected SD rats presented either ECM with ascendant paraplegia, or severe anemia associated with malaria hyperparasitemia (namely NoECM *i*.*e*. without ECM signs). Fig 1 presents the survival curves of both ECM and NoECM rats. 18 out of 40 infected SD rats (45%) exhibited ECM symptoms. ECM occurred within 5 to 9 days post infection—*pi* (median = 5.5 IQR [5; 7]) with death between day 5 and day 12 *pi* (median = 6 IQR [5; 6]). 14 among the 18 ECM rats (77.8%) showed paresis that quickly evolved to limb paralysis, and then, to global paralysis, respiratory distress, coma and death within 12 hours. The 4 remaining ECM rats spontaneously reversed their limb paralysis within 24–48 hours; one died later of hyperparasitemia and severe anemia (day 12) and the 3 others became cured with a total parasite clearance, at days 18–20 post-infection (*pi*) (S1C Fig).

The 22 other rats of the 40 infected had no clinical signs of ECM, but presented symptoms of severe malaria with discoloration of skin and eyes, strong anemia and weight loss. 19 out of 22 NoECM rats died between days 10 and 17 (median = 12, IQR [11.25; 14]) (Fig 1) and 3 remaining NoECM rats developed mild malaria and survived after total parasite clearance (S1C Fig).

Our results are close to those already reported in SD rats infected by *P*. *berghei* ANKA [28,29]. Cerebral complications including paralysis, convulsion and coma with death around day 5–8 *pi* were also observed with C57BL/6 and CBA/J mice infected by *P*. *berghei* ANKA [30,31].

These ECM manifestations in SD rats are close to the most common complications of falciparum malaria in children [2,32,33] largely observed in Africa [32–38] the continent with the most cases of this pathology in terms of morbidity and mortality. According to WHO criteria, Human CM corresponds to a severe malaria with coma [4]. However children presenting CM can develop focal neurological signs, decerebrated posturing due to raised intracranial pressure, impairment of consciousness, behavioral changes, and convulsions. Although most children with CM regain consciousness after specific treatment within 48 hours and seem to make a full neurological recovery, approximately 20% die and up to 10% have persistent neurological after-effects including lack of sensitivity, hemiplegia, and quadriplegia [2,39]. The convulsions were absent in rat ECM and even though they are frequently observed in children with CM, this symptom is rarely reported in Human CM in adults [40]. The SD rat model presented thus many clinical features common to Human CM.

### Parasitemia evolution in ECM and NoECM rats

The parasitemia in both ECM and NoECM groups was microscopically determined from day 3 *pi* and increased gradually (S1A Fig). Parasitemia of ECM rats varied from 6.3% when the first symptoms occurred to a maximum of 23% for the latest case recorded while parasitemia of NoECM rats could reach very high values (89%) leading to fatal severe anemia. One out of 4 ECM rats which spontaneously reversed their limb paralysis, died later with hyperparasitemia (47%) while the 3 other ECM rats, showed total parasite clearance at days 18–20 *pi* after the parasitemia reached 38 ± 4.5% at day 12 *pi* (S1C Fig). Three NoECM rats developed mild malaria and showed a total parasite clearance at days 16–20 *pi* after a parasitemia peak of 26.3 ± 2.1% at day 12 *pi* (S1C Fig).

**Fig 2:**
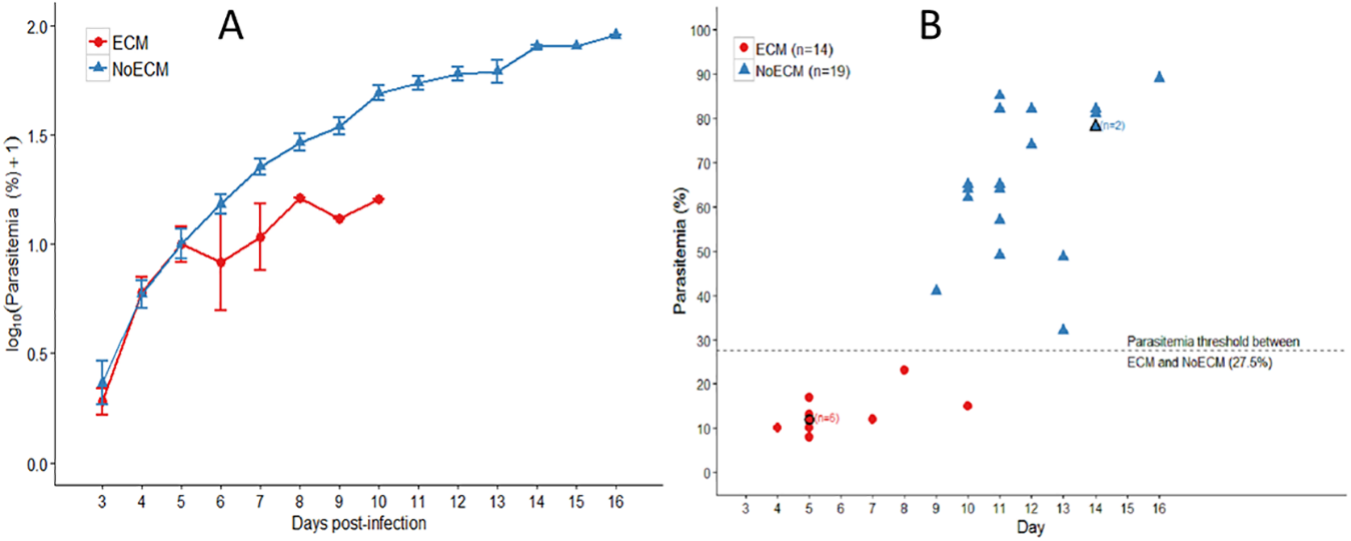
Parasitemia analysis. **A)** Parasitemia (Log%+1) in Sprague Dawley rats infected by K173. Young SD rats 4.5 weeks old were infected with 5.10^7^ parasitized mouse erythrocytes by ip injection. Parasitemia determined from Giemsa stained tail blood smears was monitored from day 3 *pi*. Parasitemia in ECM and NoECM is presented versus days post-infection. Parasitemia is expressed in mean ± standard error (SEM). **B)** Distribution of ECM (n = 14) and NoECM (n = 19) rats versus parasitemia at the day of the death. Some rats died very early on the morning and their last reported parasitemia dated from the previous evening explaining why some points have one-day difference between Fig 1 and Fig 2B. There was no overlap of the two populations leading to a parasitemia threshold of 27.5% being established allowing the prediction that over this value all infected rats will not display a CM event but severe anemia.

The difference in the course of the parasitemia between the two groups (ECM *vs* NoECM) was not statistically significant over a comparable period (Fig 2A). Fatal ECM events always occurred at a parasitemia inferior or equal to 23%. By contrast no NoECM rats died under a 30% parasitemia (Fig 2B) allowing the statistical threshold of 27.5% separating both groups. However neither the levels of parasitemia nor its kinetics are discriminant criteria for the prediction of ECM appearance in the rat model.

We can hypothesize that ECM outcome is not only dependent on the parasite virulence but also on the host SD rats. These animals are non-consanguine and each individual displays its intrinsic physiological sensitivity to the parasite. However, this SD rat model has the advantage of allowing a comparison within the same population of rats of the effects of the infection between ECM and severe malaria no ECM through the follow-up of biological, clinical and histological parameters. Even though parasitemia associated with Human CM is undocumented, according to WHO criteria, a high density of parasites in the blood (> 4%) increases the risk of deterioration to severe malaria [4]. However low parasitemia are linked with human CM occurrence [41].

### ECM in SD rat is not associated to anemia

Hematological parameters were studied for 13 control rats, 17 ECM and 22 NoECM rats. We analyzed the impact of K173 infection on red and white blood cells and platelet counts.

**Fig 3:**
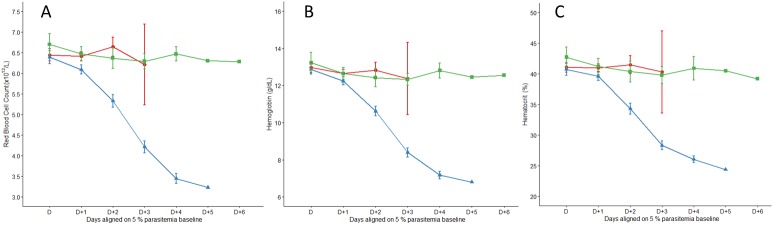
Blood parameters analysis. Red blood cells (RBCs) (**A**), hemoglobin (HGB) (**B**) and hematocrit (HT) (**C**) in ECM (n = 17), NoECM (n = 22) and control (n = 13) groups during K173 infection. The hematological parameters are aligned from and on the basis of day (**D**) where parasitemia was estimated at 5% (S1B Fig). Hemoglobin and hematocrit also displayed same trends between ECM and NoECM groups (3B and 3C). All data are represented by mean ± standard error of the mean (SEM).

**Red Blood Cells** (RBCs): The RBCs, hemoglobin and hematocrit levels were analyzed to assess anemia (Fig 3 and S1 Table). The progression of RBC counts during the course of infection (Fig 3A) showed that ECM rats presented a stable RBC count remaining at the level of the controls but was significantly different with NoECM (p = 0.036). From D+2 (2 days after the point when parasitemia was estimated at 5%) (S1B Fig), RBC count in the NoECM group decreased gradually as a function of parasitemia (r = 0.75, p<0.001). For control rats, day D corresponds to Day 5 after the beginning of the experiment. Hemoglobin (HGB) (p = 0.005) and hematocrit (HT) (p = 0.026) also displayed the same trends between ECM and NoECM groups (Fig 3B and 3C, respectively). The decrease of these 3 parameters only in NoECM rats attested to the progression towards severe anemia while the stability at the control level was associated with a high risk of ECM. This result revealed that ECM occurrence in SD rats was not associated to anemia. However Muller *et al*. [42] established that anemia was not associated with the frequency of malaria episodes in children, nor with malaria prevalence but was significantly associated with malnutrition. Indeed, malaria, anemia and under-nutrition are 3 diseases frequently co-existing in children aged less than 5 years in the sub-Saharan endemic area [43] explaining that CM in children, commonly presenting anemia [44].

**Fig 4:**
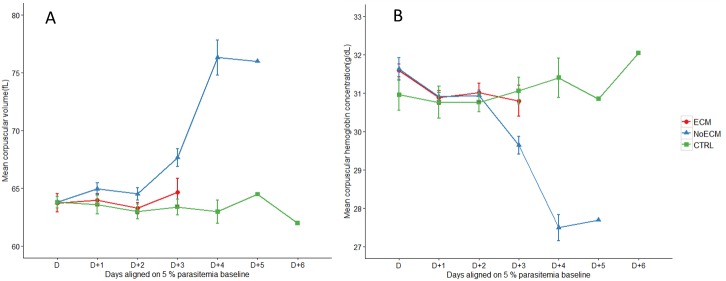
Hematological parameters in the course of infection in ECM (n = 17), NoECM (n = 22) and control (n = 13) groups. Mean Corpuscular Volume (MCV) (**A**) and Mean Corpuscular Hemoglobin Concentration (MCHC) (**B**) kinetics. The hematological parameters are aligned from and on the basis of the day (D) when parasitemia was estimated at 5% (S1B Fig). All data are represented by mean ±standard error of the mean (SEM).

The Mean Corpuscular Volume (MCV) (Fig 4A), the Mean Corpuscular Hemoglobin Concentration (MCHC) (Fig 4B), the Mean Corpuscular Hemoglobin (MCH) (S3A Fig), and the Red Cell Distribution Width (RDW) (S3B Fig) were used to characterize the type of anemia affecting the NoECM rats. In ECM rats, MCV remained stable over time of infection and comparable to control values while in anemic NoECM rats, MCV increased gradually highlighting an increase of RBC volume corresponding to a strong arrival of young reticulocytes in the blood flow (reticulocytosis) (Figs 4A and S3B). The utility of a reticulocyte count only appeared after data analysis, explaining why it was not carried out during blood analysis. However, we can assume that the MCV increase implies macrocytic and regenerative anemia. From D+3, the MCV was significantly higher in NoECM compared to control rats (p = 0.015). The MCHC values likewise remained stable in NoECM rats until D+3 but then decreased compared to control rats (p = 0.001) indicating a shift from normochromic to hypochromic anemia (Figs 4B and S3A; S1 Table).

The follow-up of anemia parameters such as MCV, MHCH, MCH and RDW were in accordance with the RBC count, hemoglobin and hematocrit levels and showed that they clearly discriminated ECM from NoECM rats (S2 Fig). Anemia is a constant feature of malaria infection. Pregnant women mostly primigravidae and children below the age of 5 years are the most afflicted. Anemia pathogenesis is multifactorial and incompletely understood. Among several factors, the destruction of erythrocytes (RBCs) and the removal of non-parasitized RBCs in acute malaria are the most frequently observed causes of severe malarial anemia [45].

**Fig 5:**
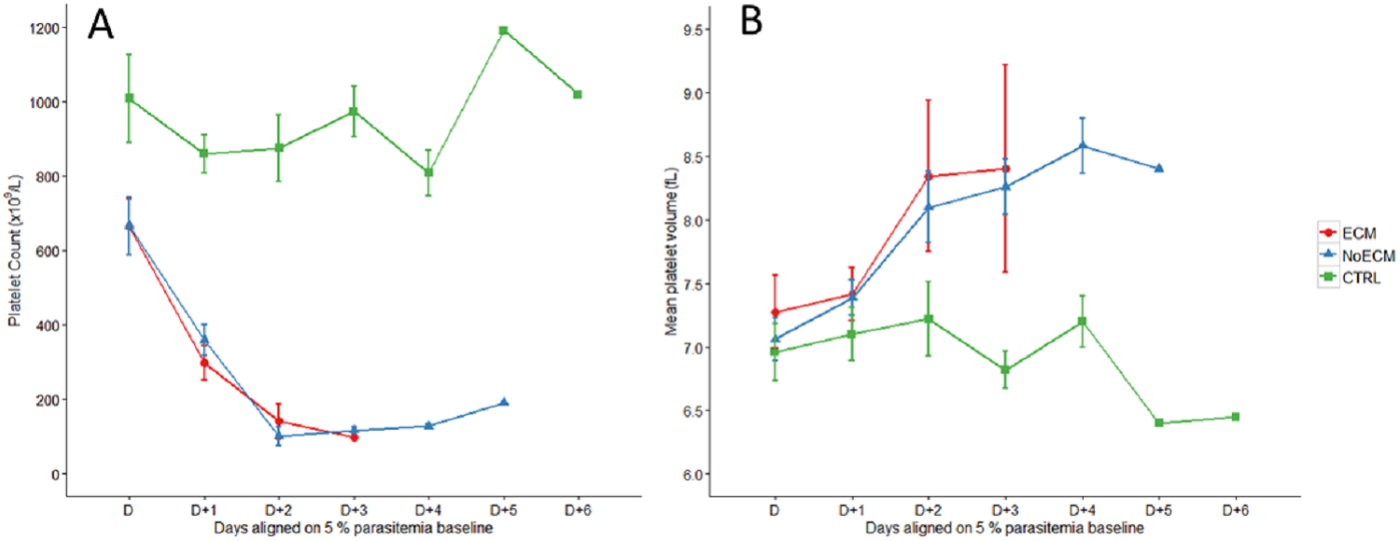
Hematological parameters in the course of infection in ECM (n = 17), NoECM (n = 22) and control (n = 13) groups. Platelet counts (**A**) and Mean Platelet Volume (MPV) (**B**). The hematological parameters are aligned from and on the basis of the day (**D**) when parasitemia was estimated at 5% (S1B Fig). All data are represented by the mean ± standard error of the mean.

**Platelets**: Platelet count (Fig 5A), Mean Platelet Volume (MPV) (Fig 5B) and the Platelet Distribution Width (PDW) (S4 Fig) were analyzed to characterize the impact of K173 infection on SD rat platelets (S1 Table). A drastic decline of platelet count was observed over time in infected rats (ECM and NoECM) compared to control (p<0.001) (Fig 5A). This thrombocytopenia was inversely proportional to the parasitemia (r = 0.82, p < 0.001) but no significant difference of platelet counts was observed between ECM and NoECM groups (p = 0.496) indicating a simple link between thrombocytopenia and K173 infection. The MPV parameter study presented a similar increasing trend in all infected groups (Fig 5B). The increased MPV values reflect the augmentation in the bloodstream of the number of young platelets, which are larger than old platelets. This indicates that the thrombocytopenia in infected rats was peripheral because it was regenerative and may thus be due to strong platelet consumption. This phenomenon is similar to platelet patterns in Human severe malaria especially in children [39,46–50].

**Fig 6:**
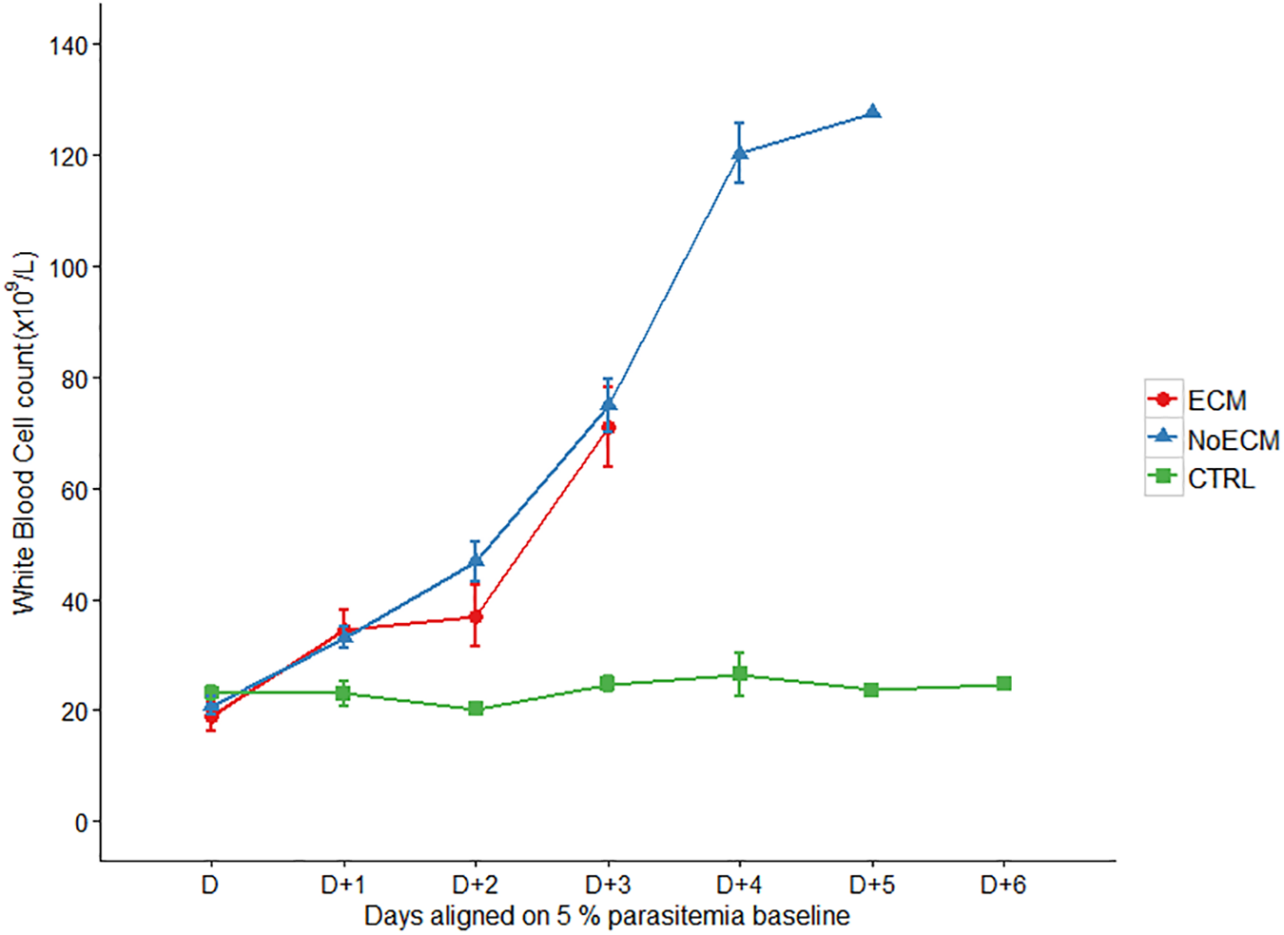
White blood cell count during the course of infection in ECM (n = 17), NoECM (n = 22) and control (n = 13) groups. Parameters are aligned from and on the basis of the day (D) where parasitemia was estimated at 5% (S1B Fig). All data are represented by the mean ± standard error of the mean (SEM).

**White Blood Cells** (WBCs): The total and differential leucocyte counts were significantly increased during K173 infection compared to the control group. This count was correlated to following the parasitemia but no difference between ECM and NoECM rats was observed (Figs 6 and S2; S1 Table). This increase mainly concerned lymphocytes (S5C Fig), granulocytes (S5A Fig) and less but still significantly monocytes (S5B Fig). This leukocytosis including lymphocytosis and monocytosis was in accordance with observations in children suffering from falciparum malaria, for whom high levels of lymphocytes were reported [50].

### ECM in SD rats leads to multivisceral deficiencies

**Fig 7:**
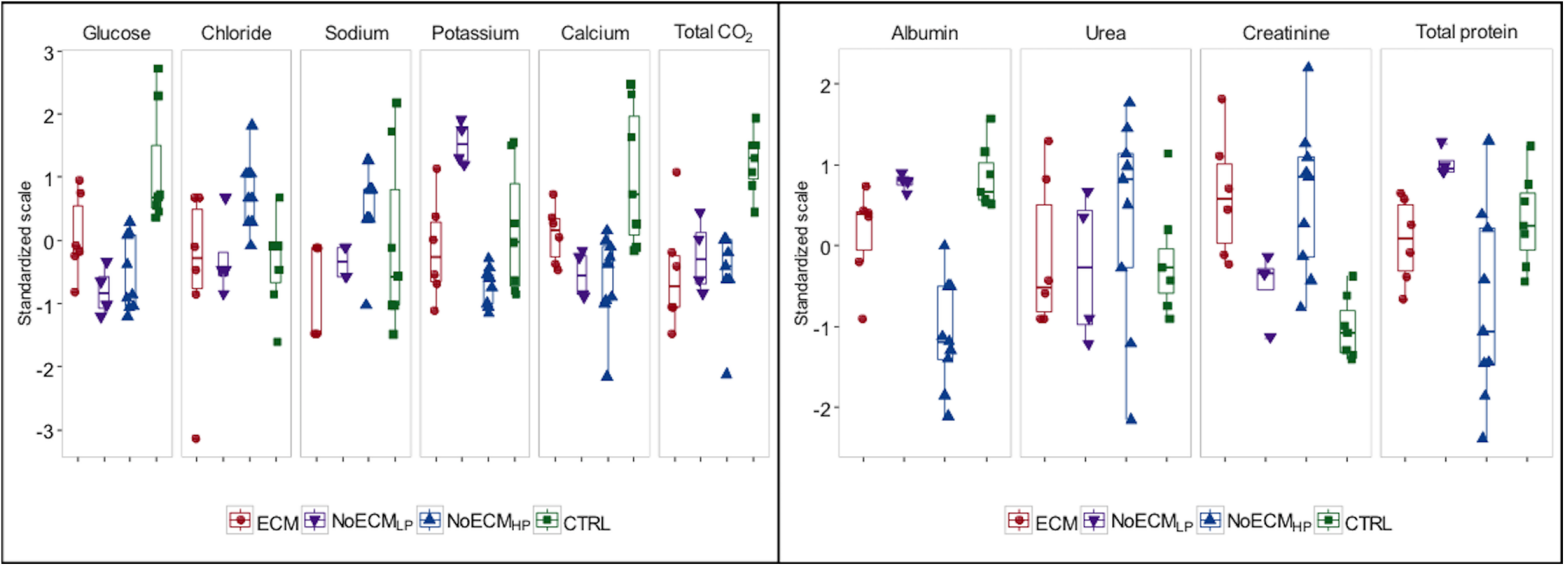
Biochemical parameters of SD rats infected by K173. Globally significant differences between ECM, NoECM and control (CTRL) groups were observed for creatinine (p = 0.001) potassium (p<0.001) glycemia (p<0.001), calcium (p = 0.002), albumin (p<0.001) and total CO_2_ (p<0.001). The ECM group was significantly different from the CTRL group for creatinine (p = 0.001), glucose (p = 0.037) and total CO_2_ (p<0.001), from the NoECM_LP_ group only for potassium (p = 0.004) and from the NoECM_HP_ group for albumin (p = 0.001) and chloride (p = 0.0029). To suppress the influence of parameter units, the data are plotted on a standardized scale, e.g. mean = 0 and SD = 1.

Because of the great variations in parasitemia in the NoECM group, the NoECM rats were split into 2 cohorts of differing parasitemia level. Group 1 had a lower parasitemia (NoECM_LP_: mean parasitemia = 26.5% ± 2.25; n = 4) close to the parasitemia of ECM rats (mean 21.8% ± 2.6; n = 6), (p = 0.218) and Group 2 presented hyperparasitemia (NoECM_HP_: mean parasitemia 61.4% ± 3.3; n = 9), (p<0.001). Among all biochemical parameters measured (Fig 7 and S2 Table), ECM rats presented a significant increase of creatinine levels (p = 0.001) and a significant decrease of glycemia (p = 0.037) and total CO_2_ (p<0.001) compared to the control group. The same was observed in NoECMs. High levels of creatinine, observed principally in ECM and NoECM_HP_ rats, have also been reported in children suffering of CM in Africa where hypercreatinemia was reported to be a risk factor of mortality [37,51]. This increase could be linked to renal failure also described in severe malaria [2,52].

A significant decrease of total CO_2_ level (p<0.001) observed in ECM rats compared to control was probably a sign of metabolic acidosis. Generally, serum bicarbonate accounts for about 95% of the total CO_2_ content; thus the measurement of total CO_2_ is an excellent estimator of serum bicarbonate [53]. The lower levels of total CO_2_ result from metabolic acidosis which is one of WHO criteria of severe malaria (plasma bicarbonate concentration < 15mmol/L) [4]. Acidosis is currently observed in CM children and is considered predictive of death [54]. However, the diagnosis of metabolic acidosis is made by arterial-blood gas analysis. The low CO2 levels in venous blood here reported is thus suggestive; for definitive diagnosis an arterial-blood gas would be needed.

The low glucose levels reported in ECM rats followed a trend similar to that of hypoglycemia observed globally in CM in children [44,55]. Indeed, hypoglycemia (blood glucose < 2.2 mmol/L) is particularly common in children less than 5 years old, especially when seizures, coma or hyperparasitemia occur. In some cases of pernicious falciparum malaria, hypoglycemia less than 0.4 g/L was associated to higher mortality rates [56]. However, as also observed in our study model, hypoglycemia is not a specific complication of CM but is found in severely ill fasted children, resulting from glycogen depletion and/or possibly impaired hepatic gluconeogenesis [57].

Albumin levels also presented a decrease for ECM rats compared to control rats. However, even though a significant difference (p = 0.001) was reported between the ECM and NoECM_HP_ groups, the same trend was observed between ECM and controls (p = 0.073). Total proteins showed the same variation as albumin. The decrease in serum albumin / total proteins indicated that severe forms of K173 infection in SD rats lead to electrolyte imbalance reflecting renal and/or hepatic failure. Hypoalbuminemia in ECM and NoECM_HP_ groups associated to hypercreatinemia in the same groups, confirmed renal impairment as reported in human severe malaria including CM [58,59].

Surprisingly, potassium was significantly higher in NoECM_LP_ rats than in ECM (p = 0.004), NoECM_HP_ and control rats. Hyperkalemia is reported in children with severe malaria especially with CM [59,60]. Hyperkalemia can complicate acidosis and can increase the risk of mortality [60]. Excess potassium levels can result from acute renal failure [59] or potassium moving out of its usual location within cells into the bloodstream including destruction of red blood cells (hemolysis). This difference between anemic NoECM_HP_ rats and NoECM_LP_ could result from a lower number of RBCs being present and elimination of circulating potassium.

Globally biochemical analysis revealed that in SD rats K173 infection could lead to acute renal failure during ECM while, in NoECM_HP_ rats, a chronic renal failure was noted which can be associated to a hepatic failure. NoECM_LP_ which is a milder form of malaria presented less renal impairment than ECM and NoECM_HP_. The impact of K173 infection in this model is close to that observed in human malaria [52,59,61,62]. Hypoglycemia, acidosis and renal failure observed in this rat model are the main WHO criteria to define severe forms of malaria [4]. The SD rat model thus appears as particularly relevant to study the impact of malaria in Human CM multivisceral deficiencies.

### Cytokine expression profile in K173-infected rat

**Fig 8:**
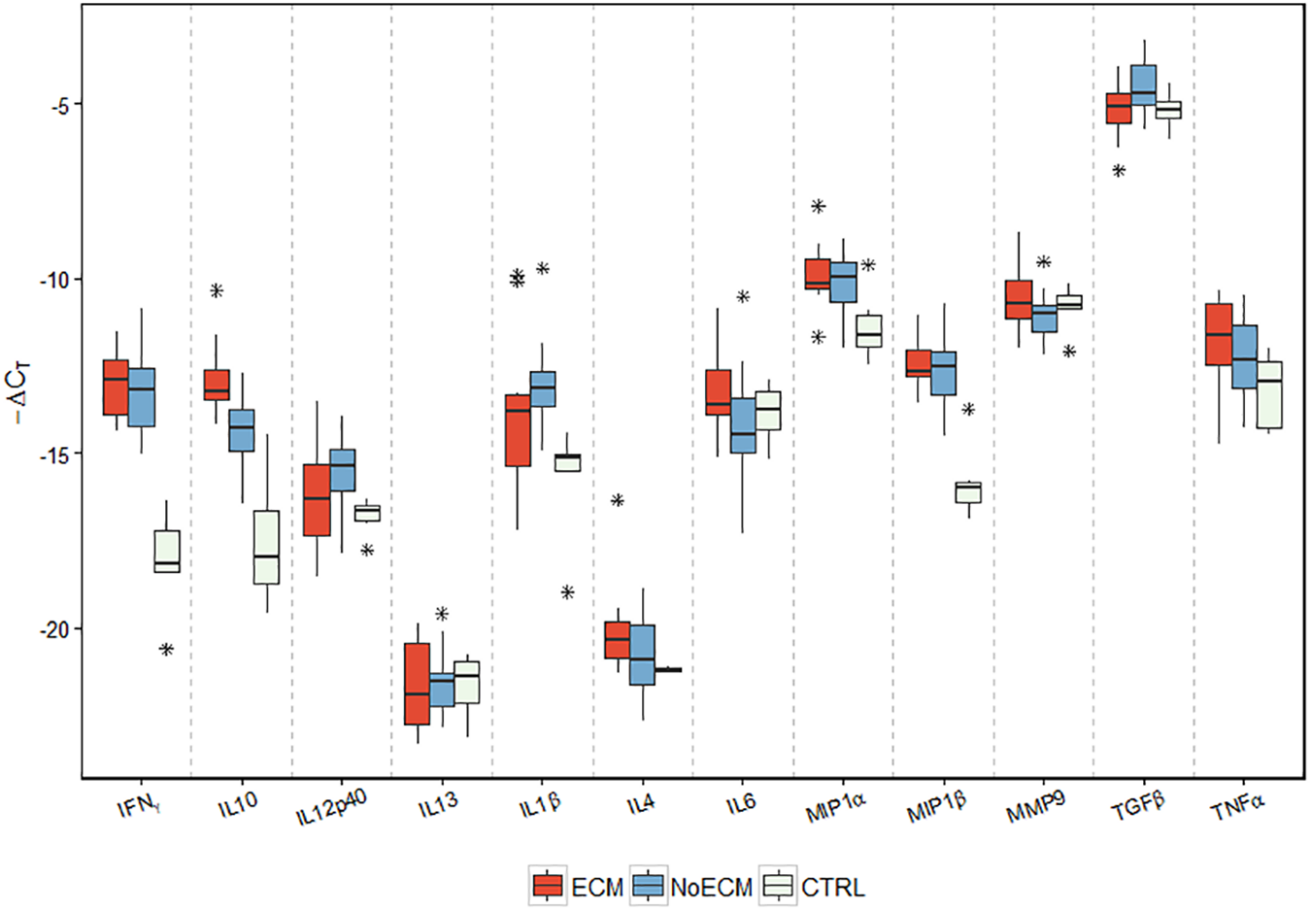
Expression of cytokines in SD rat brains infected by K173. Cerebral cytokine gene expression was assessed in ECM (n = 14), NoECM (n = 23) and Control brains (n = 7). Samples were collected on day ECM onset for ECM rats, after the parasitemia reached 30% for NoECM rats and for control rats at the same time as ECM and NoECM groups. Box plots represent medians and 25th and 75th percentiles. Among all the cytokines investigated only the gene expression of INFγ (p<0.001), IL10 (p<0.001), IL1β (p = 0.001), TNFα (p = 0.045), MIP1α (p<0.014) and MIP1β (p<0.001) were significantly different between infected and control rats. The symbol * indicates outliers.

For a better understanding of the cytokine profiles of ECM in SD rats infected by K173, brain cytokine gene expression was analyzed in ECM (n = 14), NoECM (n = 23) and Control (n = 7) brains. Among the 12 brain cytokine gene expressions assessed, INFγ (p<0.001), IL10 (p<0.001), MIP1β (p<0.001), IL1β (p = 0.001), TNFα (p = 0.045), and MIP1α (p<0.014) were significantly up-regulated in all parasitized rats compared to the control group (Figs 8 and S6; S3 Table). IL12p40 mRNA was significantly up-regulated only in the NoECM group compared to the control group (p = 0.04). Comparison of ECM with NoECM rats showed that IL10 mRNA levels were up-regulated more in ECM rats than in NoECM rats (p = 0.044) and TGFβ mRNA expression had a greater trend towards up-regulation in NoECM than ECM groups (p = 0.073). The study of IL10:TNFα level ratios showed that severe malaria cases (grouped data from both ECM and NoECM rats) are significantly lower than controls (p = 0.001). These data from cerebral cytokines are in accordance with plasma level ratio already reported in Humans [63].

The overexpression of TNFα, IFNγ, IL1β, MIP1α and MIP1β genes in both ECM and NoECM rats suggest that the variations of these cytokines were linked to the infection. MIP1α is involved in acute and chronic immune response against *P*. *falciparum* and its high levels may also cause anemia [64]. Local production of TNFα mRNA in the central nervous system plays a role in fatal murine ECM pathogenesis [65,66]. In Humans, a higher level of local production of TNFα mRNA in the central nervous system is associated to CM in children [67]. The involvement of TNFα in association with pro-inflammatory cytokines like IL1β and TGFβ was also reported in the Human CM immune response [68]. Decreased expression of TGF-β associated with increased IFNγ expression has been reported in ECM in CBA/J mice [65].

Higher levels of IL10 have been previously reported in cerebral and severe human malaria compared to mild malaria [69,70]. IL10 is an immunoregulatory cytokine that has a critical role in downscaling the immune response to pathogens to prevent host damage and high expression levels of IL10 may induce immunodeficiency as found in some parasitic and retroviral infections in humans [71]. Conversely, IL10 seems to have a neuroprotective role in ECM in mice [72–74].

Brain cytokine gene expression in the present study was not significantly different between ECM and NoECM rats except for IL10. Nevertheless, the cytokines involved in this rat model (INFγ, IL10, TGFβ, IL12p40, IL1β, TNFα, MIP1α, and MIP1β) were similar to those reported in the immune malaria response [67,68] and in ECM mouse malaria [65,66].

### Brain vascular permeability is altered during ECM in SD rat

**Fig 9:**
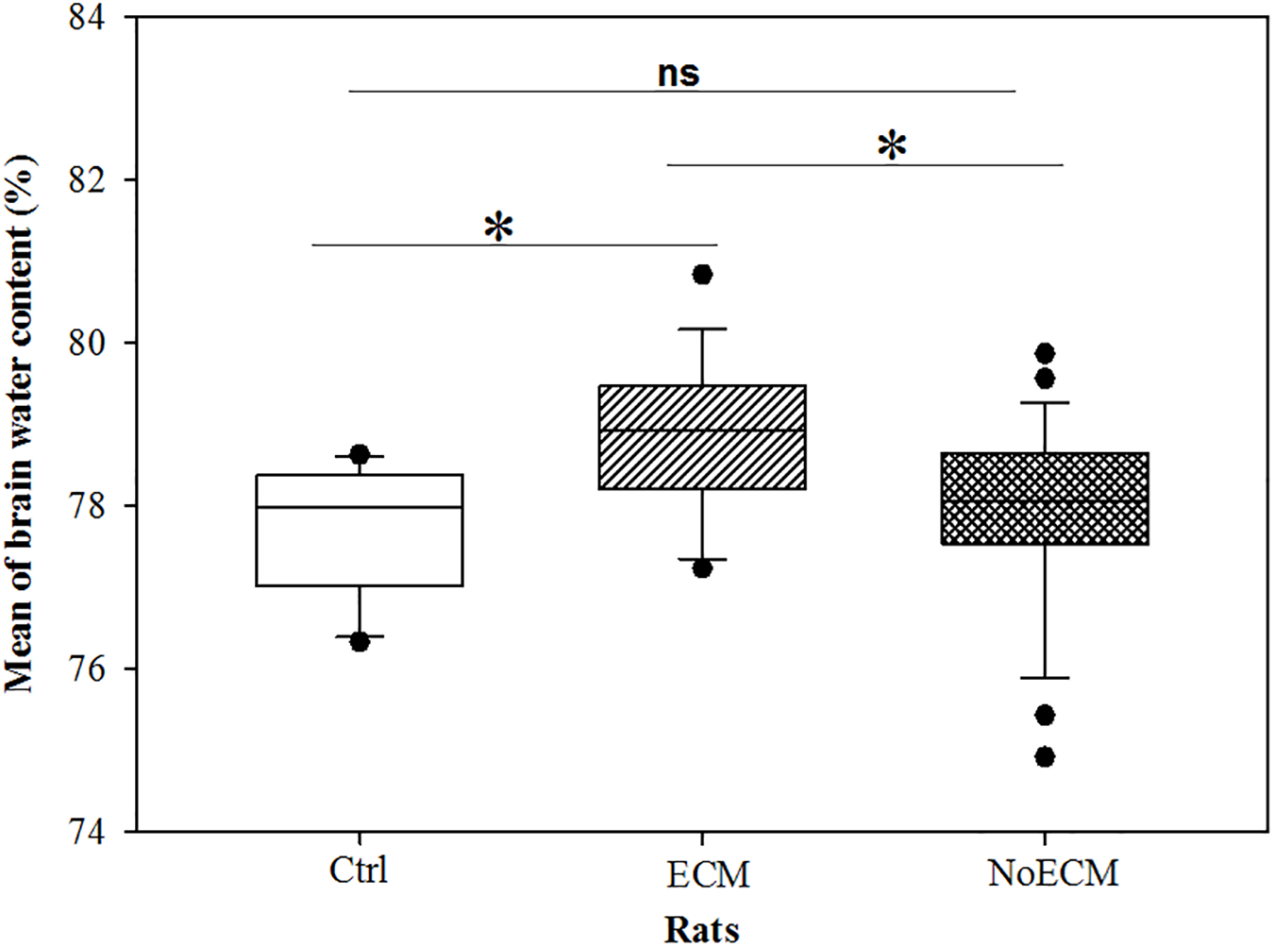
Edema test. Quantification of water contained in brains of ECM (n = 12), NoECM (n = 13) and Control brains (n = 8). The brain water content in the ECM group was statistically higher than that in NoECM (p = 0.007) and Control rats (p = 0.02). The p value was not significant (ns) between NoECM and Control rats (p = 0.66). Box plots represent medians and 25^th^ and 75^th^ percentiles. Statistical test used: Kruskal-Wallis One Way Analysis of Variance on Ranks and Donn’s Method for all pairwise comparisons. *p<0.05.

**Fig 10:**
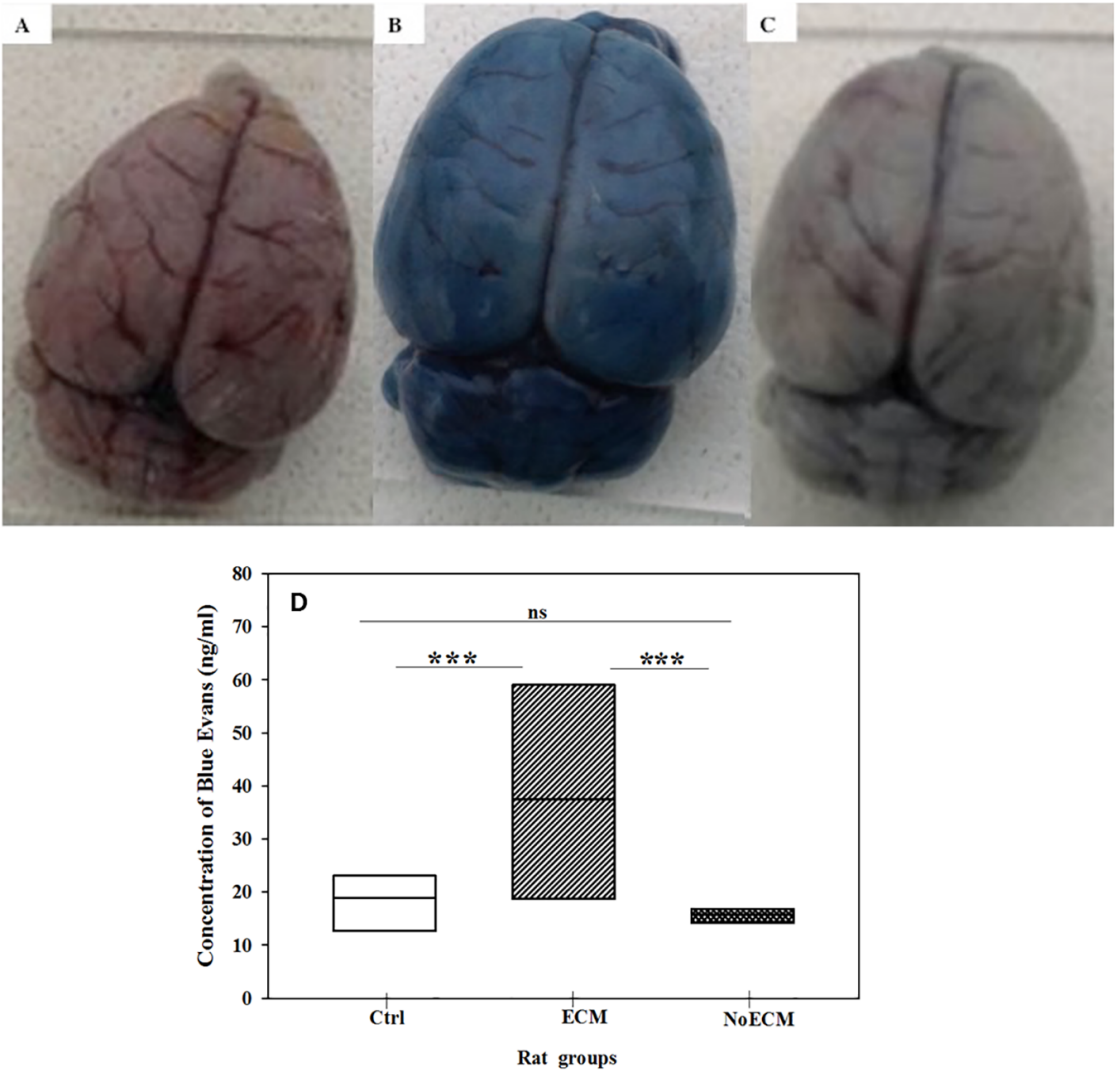
Assessment of brain vascular permeability. (**A**) Dorsal view of brain from Control (Ctrl), (**B**) Dorsal view of brain from ECM, and (**C**) Dorsal view of brain from NoECM rats after injection of Evans blue dye in tail vessel and sacrifice 90 minutes later. (**D**) Quantification of Evans blue dye concentration in brain of non-infected (Control; n = 7) and infected rats (ECM; n = 5 or NoECM; n = 8). After Evans blue dye injection, brain was removed and immersed in a 2 ml of paraformaldehyde (PFA) for 48h at 37°C. The extravasated dye was quantified by spectrophotometer. Box plots represent medians and 25^th^ and 75^th^ percentiles. Statistical test used: Kruskal-Wallis One Way Analysis of Variance on Ranks and Donn’s Method for all pairwise comparisons. *p<0.05; **p<0.005; ***p<0.001.

Autopsies of ECM rats showed brain swelling compared NoECM and controls. The brain water content in the ECM group was higher than in NoECM (p = 0.007) and controls (p = 0.02) groups (Fig 9). No significant difference was noted between NoECM and Control rats (p = 0.66). The brain swelling and the increased water content indicate altered vascular permeability. These data were confirmed by the analysis of a significant difference in tissue extravasations of Blue Evans dye between in ECM, NoECM and control brains (p = 0.028) (Fig 10B, 10C and 10A, respectively). More Evans Blue was detected in brains from ECM rats than NoECM meaning an increase of the blood brain barrier (BBB) permeability during ECM (Fig 10D). This result supports the role of BBB loss of integrity in the cerebral edema.

Brain swelling, also reported in the ECM mouse model [75–77], is a common feature of Human CM in adults [78–80] and African children [36,81–84]. The computed tomographic scans of Human CM children with severe intracranial hypertension often showed ischemic lesions [85,86]. The mechanisms responsible for the dysfunction of the BBB in Human CM have not been completely elucidated but adhesion of sequestered infected red blood cells (iRBCs) to cerebral endothelium has been proposed as a contributing factor [80,86]. Moreover the role of cerebral edema in the pathophysiological features and the clinical outcome of Human CM is not well understood and remains controversial [78,87]. Indeed, autopsies of adult Human CM patients have shown that brain swelling is not always present which does not support a direct link between brain swelling and a fatal outcome [78,88].

### ECM in rats is associated to brain hemorrhages and sequestration in cerebral microvasculature

**Fig 11:**
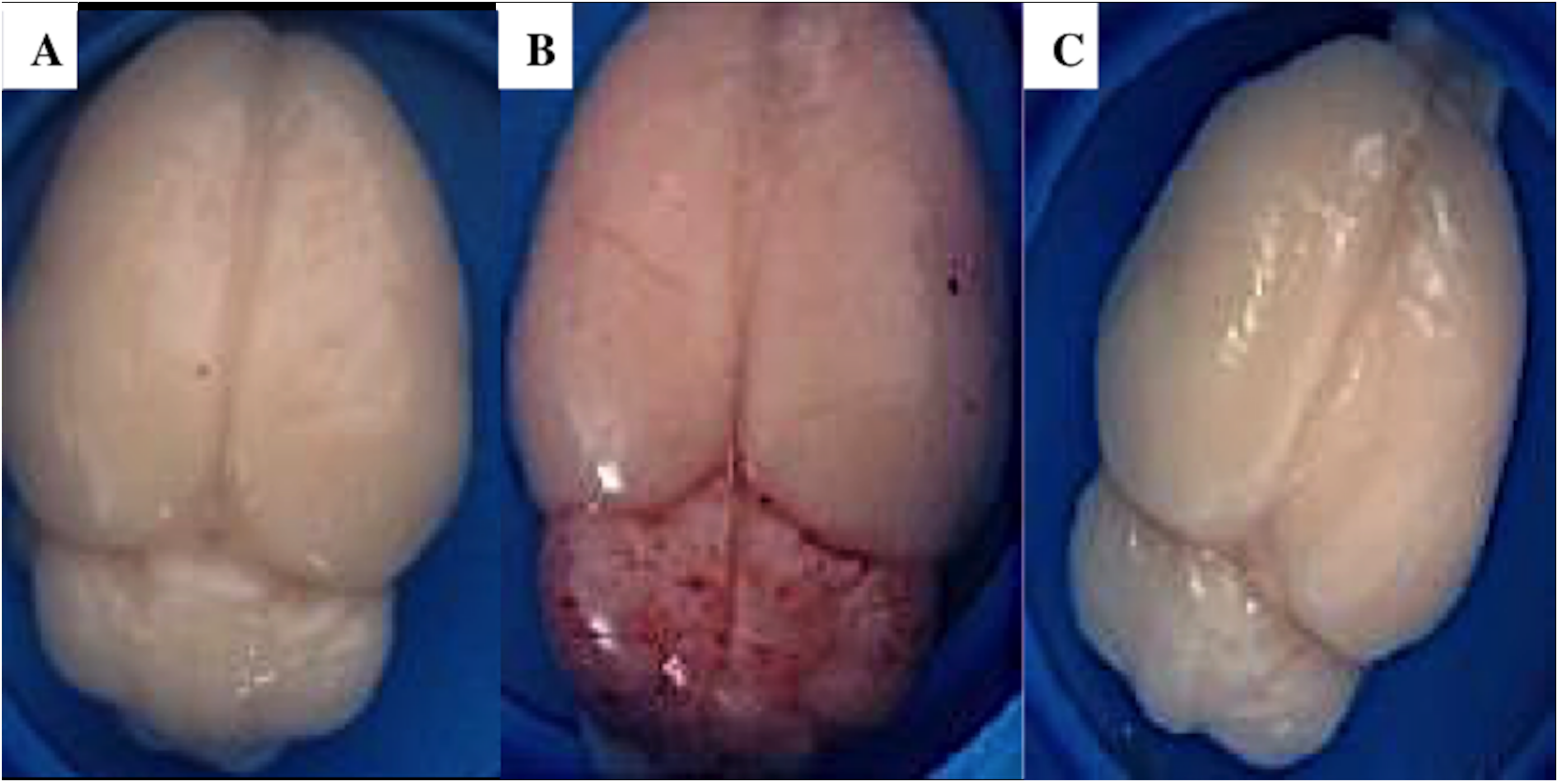
Dorsal view of brains from K173 infected SD rats after systemic lavage. (**A**) Healthy brain of a Control (Ctrl) rat. (**B**) ECM brain shows petechial hemorrhages especially on cerebellum and edema with disappearance of cerebral longitudinal fissure. (**C**) NoECM brain presents no macroscopic lesion.

Gross examination of ECM (n = 12) brains showed an increased volume (ventriculomegaly) which is an obvious sign of brain swelling and petechial hemorrhages under the meninges on both cerebral hemispheres, more especially on the cerebellum (Fig 11B). By contrast, brains of NoECM rats (n = 16) had no macroscopic lesions (Fig 11C). The lesions are similar to those reported in autopsies of Human CM patients including a grey discoloration of the brain substance, petechial hemorrhages, predominantly in the white matter and cerebellar folia [2,38,86,89]. Before systemic lavage, NoECM rats showed no brain hemorrhagic lesion but a discoloration, sign of symptomatic severe anemia, in comparison with control and ECM rats. After systemic lavage, No-ECM brains were comparable to Controls (Fig 11A) with no observed lesions.

**Fig 12:**
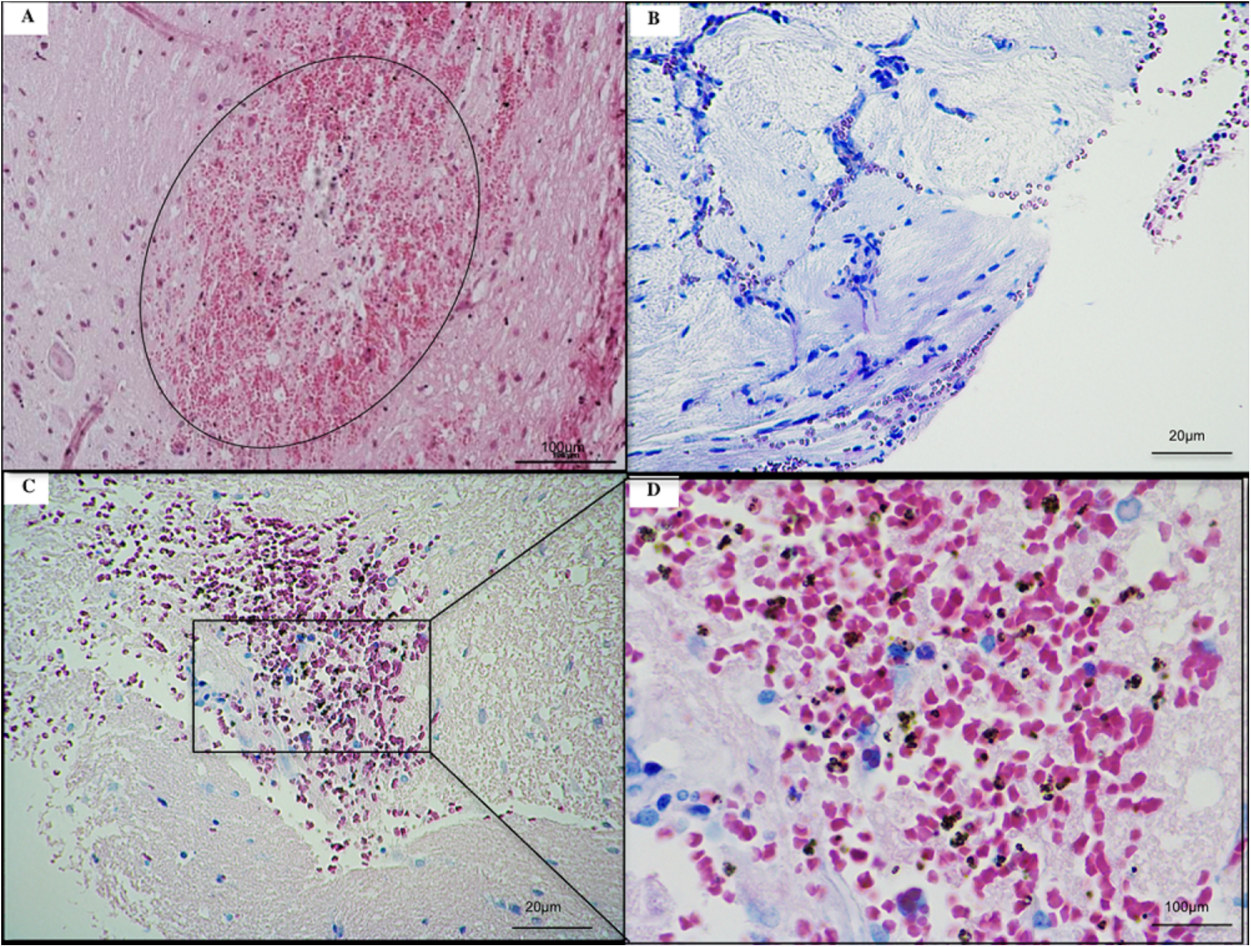
Histological illustrations of cerebral lesions in ECM K173 infected SD rats. (**A**) Acute perivascular neuropilar hemorrhage with severe extension (circle). Diffuse intracerebral hemorrhages (**B** and **C**) with presence of numerous parasites (**D**). ECM brains were collected from rats without systemic lavage. Histological slices of ECM brains stained using hematoxylin-eosin.

**Fig 13:**
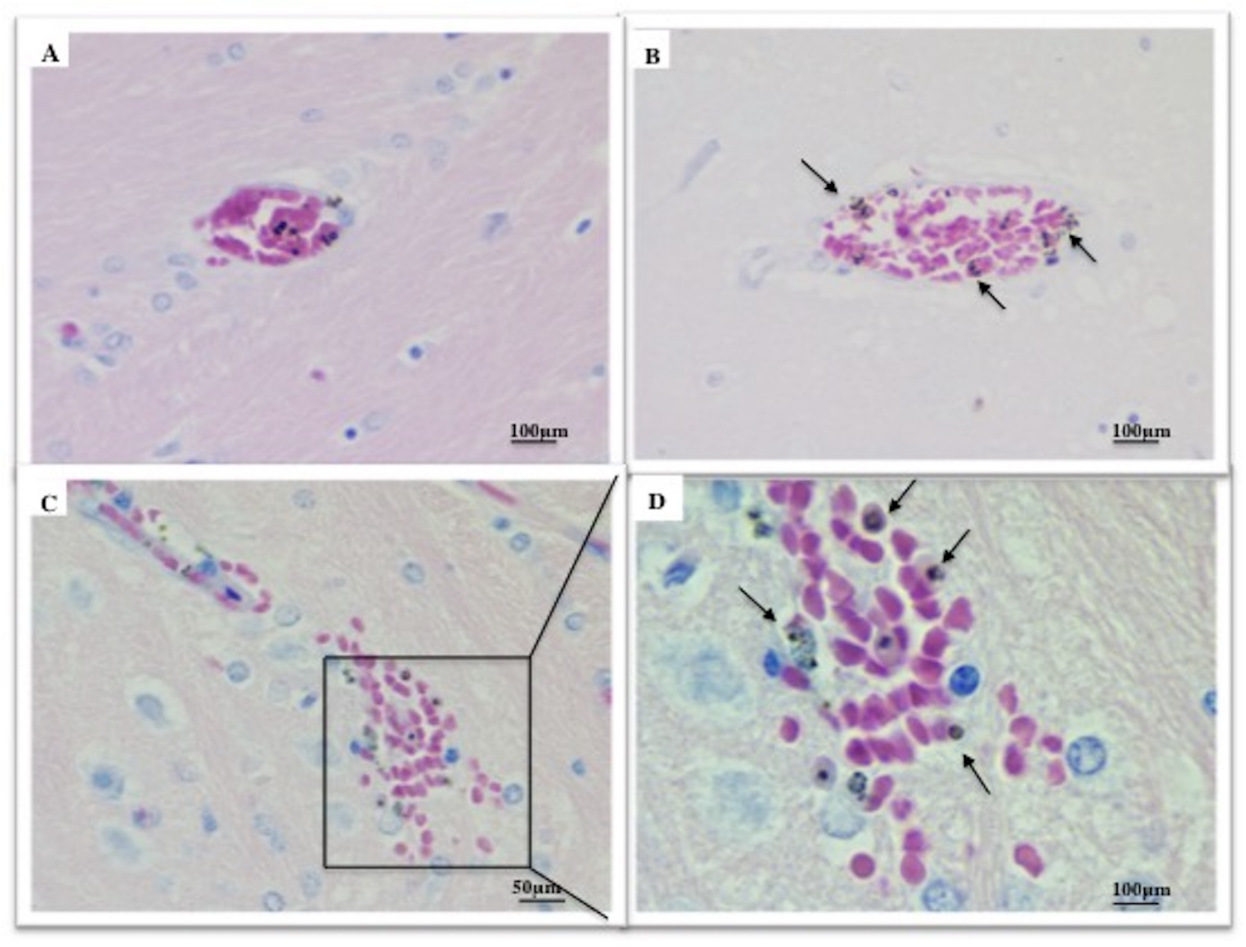
Localization of parasites in cerebral vessels in ECM K173 infected SD rats. (**A**) Thrombosis. (**B**) Parasites were localized in peripheries of cerebral vessels in direct contact with the endothelium. **(C** and **D)** Extravascular hemorrhage with parasites (arrows) localized in peripheries. ECM brains were collected from rats without systemic lavage. Histological slices of ECM brains stained using hematoxylin-eosin.

**Fig 14:**
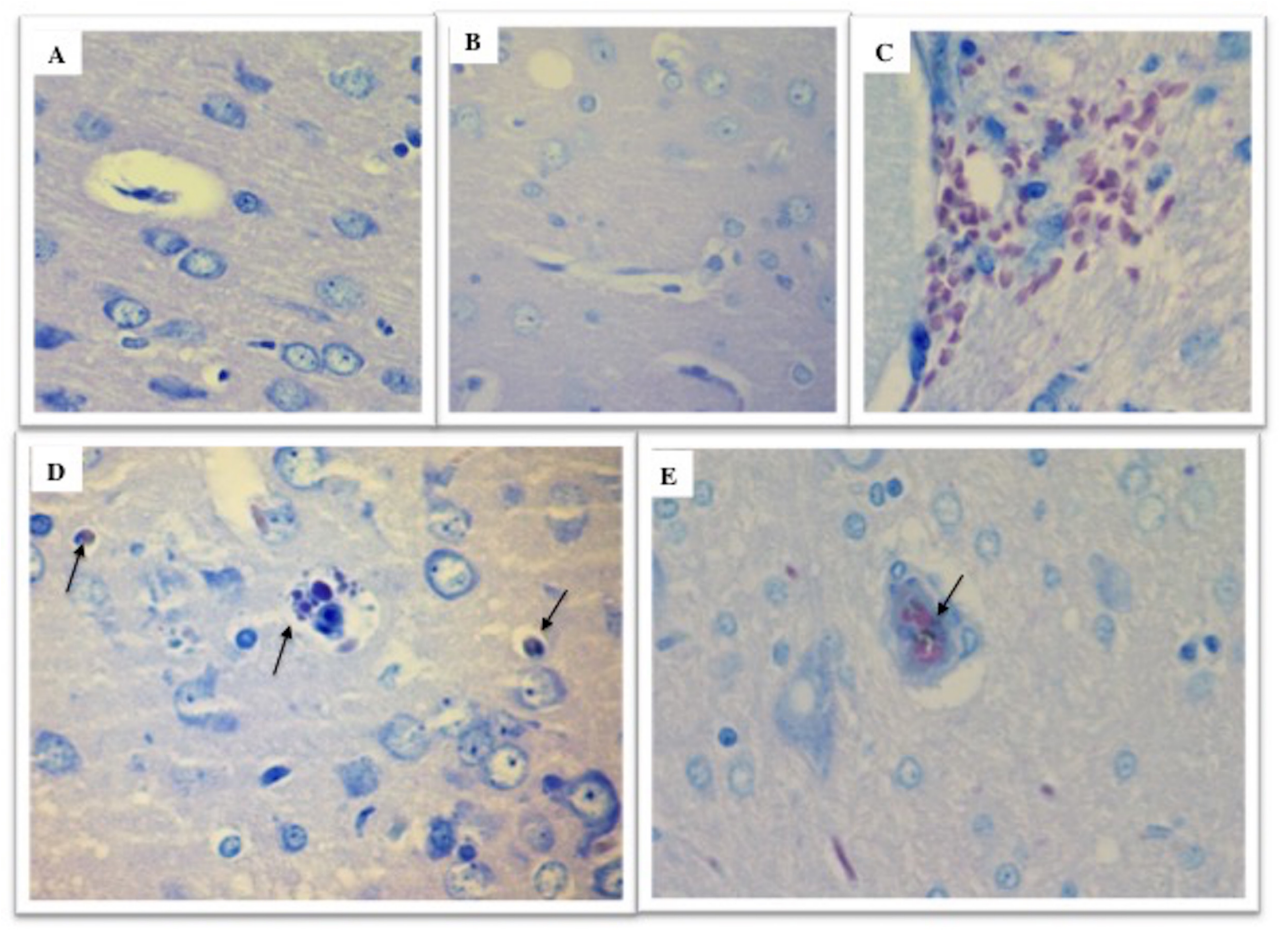
ECM in SD rats is associated with sequestration of infected red blood cells. Systemic lavage cleans out blood in micro-vessels. Parasites and lesions were totally absent in micro-vessels of Control (**A**) and NoECM brains (**B**). However in ECM brains, hemorrhagic lesions persist (**C**) and parasites were still present in micro-vessels (arrows) (**D** and **E**). Histological slices of ECM brains stained using hematoxylin-eosin.

In the ECM group, hemorrhages were mainly observed in brainstem and cerebellum on peri-meningeal and peri-ventricular areas in white and gray matter (S5 Table). The histological slices of ECM brain showed acute perivascular and neuropilar hemorrhages with severe extension associated with interstitial edema (Fig 12A). The hemorragic lesions were severe and extended in brain matter (Fig 12B). Numerous parasites were observed in hemorrhagic lesions (Figs 12C, 12D, 13C and 13D). Thrombosis was present in meninges and parenchyma (Fig 13A). Gliosis and neurophagia, with varying degrees of severity, were also observed in ECM and NoECM rats (S5 and S6 Tables). A large deposit of iRBCs was observed in micro-vessels of all infected rats mainly in NoECM rats (S5 Table). In ECM brains, parasites were particularly localized in direct contact of vessels walls raising the question of sequestered parasites (Fig 13). A systemic brain lavage to drain out blood stasis allowed us to clearly report parasite sequestration (intravascular and/or adhesion in micro-vessels) in all ECM brains (Fig 14D and 14E and S6 Table). By contrast, no macroscopic lesions were noted and iRBCs were totally absent in micro-vessels for any NoECM brains (Fig 14B) as Control brain (Fig 14A) after brain lavage, and despite hyperparasitemia in excess of 30%.

Before systemic lavage, NoECM rats showed no brain hemorrhagic lesion but a discoloration, sign of symptomatic severe anemia, in comparison with control and ECM rats. After systemic lavage, NoECM brains were comparable to Controls with no observed lesions.

These results suggest that the sequestration of iRBCs in cerebral micro-vessels, considered as one of the main factors of Human CM occurrence [2,36,89], is observed in all ECM rats in the present model. Moreover, washing of the blood circulatory system is a robust technique which enables removing all ambiguity between adherent iRBCs and iRBCs in blood stasis. Therefore the present ECM rat model seems a relevant model of human CM. In ECM mouse models, even though iRBC sequestration can be reported, sequestered cells are essentially leukocytes [13,90,91,92].

## Conclusion

The present experimental model of cerebral malaria in young Sprague Dawley rats infected by K173, appears as a particularly relevant model regarding the histological, physiological and biochemical parameters compared to those observed in Human CM. The most remarkable point is the sequestration of iRBCs in brain micro-vessels in all ECM rats like in Human CM whereas this phenomenon is debated in the mouse model [13,93]. In this rat model, hematological parameters susceptible to be predictive of the ECM progression were also determined. The parameters most affected in the ECM group were platelets and white blood cells with regenerative thrombocytopenia and leukocytosis as observed in Human CM while RBC count remained normal. It would be of great interest to determine whether the RBCs associated with hematocrit and hemoglobin, during the hospitalization of patients affected by malaria could be used to predict severe outcomes. In Human CM, it is urgent to stop and to reverse the development of symptoms, as quickly as possible, because of the high risk of neurological sequels. Even though artemisinin-based therapies decrease parasitemia rapidly, the neuropathology also needs to be treated, perhaps by an adjunct therapy associated to the antiparasitic compound. For the moment none of the compounds tested (for instance: protein C, ADAMTS 13 protease activator, nitric oxide, angiotensin-II inhibitor, erythropoietin) has a real protective action against the deleterious effects of Human CM [4]. The present ECM rat model is thus an interesting tool in the search for such molecules in a relevant pharmacological approach.

## Materials and methods

### Ethics statement

All animal care and experimentation was performed according to European regulations (directive 86/609/EEC revised by the new directive 2010/63/UE) and approved by the French Institutional Animal Experimentation Ethics Committee (approvals MP/02/02/01/08, MP/03/02/01/08, MP/01/35/04/12, and 2016041515469392). The experiments were carried out in the animal facility of the Rangueil Hospital Department of Parasitology, Toulouse (France), under the control of the National Veterinary Services (accreditation N° B31 555 03). The staff in charge of the animal experiments received appropriate training and were granted a license delivered by the French Agricultural Ministry for experimentation on small laboratory animals.

### Parasites

*Plasmodium berghei* Keyberg 173 (K173) murine strain of *Plasmodium*, was maintained in 6-week-old Swiss mice. The mice received 100 μl of infected whole blood by intraperitoneal (ip) injection. Every five days, a new transfer of K173 parasites was performed from infected mice to healthy mice by the same process. K173 was transferred to rats between the 5^th^ and the 25^th^ mouse transfer [28].

### Rats and infection

Male Sprague Dawley (SD) rats 4.5 weeks old were selected as animal model. They were supplied by Janvier Labs (Le Genest, Saint-Isle, France) placed 2 per cage and maintained under Specific Pathogen Free (SPF) conditions before experiments. The animals were acclimatized for 8 days and adapted to an artificial day-night rhythm of 12 h. Food and water were available *ad libitum*.

SD rats were inoculated at 4.5 weeks old by ip injection of 5.10^7^ iRBCs collected from infected mice and diluted in 200 μL of saline solution. From the 3^rd^ day post-infection (*pi*), the monitoring of rats was performed every two hours (from 6:00 am to 10:00 pm) and all clinical signs were recorded.

Seven independent experiments were carried out for this study. The first was to evaluate parasitemia and clinical evolution and to define ECM and NoECM status, the second was to follow the hematological parameters and the third to study the biochemical variations. The immunological profiles of infected rats were also studied through by following cerebral cytokine gene expressions in two independent experiments. The impact of ECM in brain vascular permeability was evaluated by two other experiments: Evans blue test and edema test. Histopathological studies were performed to evaluate sequestration phenomena at the same time as cerebral cytokine evaluation.

### Assessment of clinical evolution and definition of ECM and NoECM

To determine the survival rate and objective criteria for euthanasia to reduce animal suffering, 40 infected rats in two independent experiments were monitored without preliminary human intervention in order to follow clinical and parasitical evolution in a reference experiment of this experimental disease. However, during the entire experiment, a high vigilance about animal suffering problems was set up in order to minimize it. Each potential pain situation was assessed and kept to a minimum with two strict humane endpoints: parasitemia of rats superior to 80% and rats presenting coma. This preliminary experiment permitted to affine humane endpoints and then to euthanize animals earlier avoiding animal suffering. From 18 rats with ECM symptoms, 14 died without euthanasia because of the quick evolution of the disease and death within 12 hours.

The clinical progression in rats was evaluated by neurological assessment based on the animals’ mobility. A diagnosis of ECM was made when infected rats presented neurological symptoms characterized, firstly, by paresis, followed by limb paralysis (right and/or left) and then total paralysis before coma. Data from this survival assay allowed accurate separation of a NoECM group, for animals without ECM, *i*.*e*. rats without any neurological signs but presenting parasitemia of over 23%. For analyses requiring the death of the rat (histopathological examinations, Evans blue dye assay, cerebral cytokine analysis and cerebral edema test), the rats were sacrificed in a CO_2_ chamber at the presumed onset of objective symptoms of ECM or NoECM according to the above-mentioned definition. For assays with longitudinal monitoring (hematological, biochemical), classification into the ECM or NoECM goups was carried out *a posteriori*, according to the above definition.

### Assessment of parasitemia

Parasitemia was determined daily from day 3 *pi*, by microscopic examination of tail blood smears after Giemsa staining. The kinetics of parasitemia was not only modulated by the time (*i*.*e*. days post-infection) but also by the inter-individual variability of the rats. Before 5% parasitemia, this kinetics of parasitemia varied greatly from one animal to another. Some rats reached 5% parasitemia four days *pi* while for others it took 6 to 8 days *pi*. After reaching 5% parasitemia the progression of parasitemia was similar for all infected rats. As the clinical status of infected rats may depend on the level of parasitemia, the results were analyzed not as a function of days post-infection but rather from a baseline set at 5% of parasitemia (day D) (S1B Fig). For control rats, the Day D corresponds to Day 5, *i*.*e*. the mean of the day D for 5% parasitemia after the beginning of the experiment.

### Hematological parameters analysis

A 30 μL-tail blood sample was taken from on each rat, 4 hours before infection, and then daily until day 3 *pi*. The blood was collected through micropipettes (Microvette^®^, K2 EDTA, Sarstedt, France) and analyzed within 15 to 60 minutes. Daily hemograms were followed up during the timescale of the experiment. Estimation of hemoglobin content, hematocrit, red blood cell (RBC), white blood cell (WBC) and platelet count were performed by the hematologic analyzer (ABX MICROS-60-Diagnostics, Horiba ABX SAS, France). The differential WBC counts include granulocytes, lymphocytes and monocytes. Mean cell volume (MCV) indicates the volume of the average red blood cell and is expressed in femtoliters (fl). Mean cell hemoglobin (MCH) represents the absolute amount of hemoglobin in the average red blood cell in a sample and is expressed in picograms (pg) per cell. The MCH was calculated from the hemoglobin and the RBC data. Mean corpuscular hemoglobin concentration (MCHC) is the average amount of hemoglobin per deciliter of red cells (g/dl). The red blood cell distribution width (RDW) is a calculation of the variation in the size of RBCs. The mean platelet volume (MPV) was also measured.

### Biochemical analysis

Animals were anesthetized with isoflurane and the blood collected from the retro-orbital sinus in dry plain tubes. Blood collection on ECM rats was completed as soon as clinical signs occurred. For NoECM the collection was carried out *a posteriori*, as defined above. The blood sampling was done on control rats at the same time as ECM and NoECM rats. Biochemical parameters including glucose, protein, albumin, creatinine, urea, total CO_2_, sodium, potassium, chloride and calcium were measured on infected and control rats on the same blood samples by the Laboratoire Central de Biologie Médicale (LCBM)—ENVT, Toulouse, France.

### Brain quantitative real time PCR

Quantitative real-time PCR (RT-qPCR) was performed to determine the relative mRNA expression levels of TNFα, TGFβ, IFNγ, MIP1α, MIP1β, MMP9, IL1β, IL4, IL6, IL10, IL12p40 and IL13 genes in brain samples collected from rats ECM, NoECM and controls. Rats were euthanized by CO_2_ at the presumed onset of objective symptoms of ECM or NoECM, brains were removed and immediately frozen in liquid nitrogen. Total RNA was isolated using Trizol reagent (Gibco-BRL, pays). RNA purification and DNase treatment were performed using respectively RNA easyminikits and DNA-free kits (Qiagen, Netherlands), according to the manufacturer’s instructions. RNA concentration was evaluated by spectrophotometry at 260 nm. Reverse transcription of RNA was performed from 2 μg for each sample using an iScript cDNA synthesis kit (Bio-Rad Laboratories, pays). Real-time PCR assays were performed on 10 ng cDNA (RNA equivalent) in 25 μL volume reaction per well using Power SYBR® Green PCR Master Mix as reporter dye, and the automated photometric detector ABI Prism 7000 Sequence Detection System for data acquisition (Applied Biosystems, France). The primers used are listed in S4 Table. The geometric means of the threshold cycle (C_T_) values of housekeeping genes (GAPDH) were used as the baseline for comparing the ΔC_T_ value [94].

### Assessment of brain vascular permeability

**Brain Water content** [95]: a cerebral edema test was performed to assess the occurrence of edema during ECM. Rats were euthanized by CO_2_ when clinical signs of ECM were observed. The brain was carefully removed, and weighed before and after being dried in a desiccating oven at 50°C for 72 h to obtain the dry weight. The same protocol was applied to NoECM and Control brains. The brain water content was calculated as follows:

Watercontent(%)=[(wetweight-dryweight)x100]/wetweight.

**Evans blue assay** [96] was performed to evaluate the integrity of the BBB during ECM. 500μl of Evans blue dye (4%) was injected into tail veins of rats, once the clinical signs of ECM were observed. After 1 hour and half, rats were sacrificed by CO_2_ and their brains collected and immersed in paraformaldehyde 4% at 37°C to extract the Evans blue dye. After 48h, the absorbance of the supernatant was measured in triplicates (100μl/well) at a wavelength of 630 nm using a BioTek ELISA spectrophotometer (Winooski, USA). Evans blue dye concentration (μg/ml) was calculated using a calibration curve. In parallel, brains of NoECM and control rats were collected and treated with the same protocol.

### Autopsy protocol and sample collection

Infected rats were sacrificed once clinical signs of ECM were observed or when the parasitemia reached 30% for NoECM rats. The rats were divided into 2 groups. In Group 1, the rats were anesthetized by muscular injection of a mix of Ketamine^®^ and Xylazine^®^ and then, after exsanguination, perfused by an intracardial injection of 180 mL of physiological serum to drain the systemic blood (BL for brain lavage) before removing the brains. In the second group, the rats were euthanized with CO_2_ and the brains removed without lavage (NBL for No Brain Lavage). Macroscopic and histological examinations and immunological analysis were performed on both ECM and NoECM rats. Parasite adherence to brain micro-vessels was also evaluated.

### Histopathological analysis

Collected brains (BL and NBL) were fixed in 4% paraformaldehyde and embedded in paraffin. Samples were dehydrated in Tissue processor HMP 110 (Microm Microtech, France), for 16 hours. Brain sections of 3–4 μm were performed with a microtome, dried at 37°C for 30 minutes and stained with hematoxylin-eosin for microscopic examination. The sections were examined for hemorrhages and inflammation. Lesions were analyzed blind and classified from absence of lesion (-) to severe injury (+++++) according to their degree of severity.

### Statistical analyses (S1 Text)

On longitudinal assessments over the days, a 2-way analysis of the variance (or covariance in the case of baseline adjustment) with repeated measurements was performed. For end-point analyses, 1 or 2-way Analysis of Variance were used. *Post-hoc* tests for pairwise comparisons were performed with Tukey’s adjustment for multiplicity or Dunnett’s adjustment for comparison to a referent group. Log transformation was applied for parameters underlying a Log-Normal distribution. As complementary analysis, Partial Least Square discriminant (PLS-DA) analyses were produced to describe the correlation structure of parameters dependent on the ECM/NoECM outcome. Data analyses were performed using “R: A language and environment for statistical computing” (R Core Team (2016)) and SAS/STAT® software 9.4 (SAS Institute Inc.).

For the Evans Blue and edema tests, the collected data were analyzed using SigmaPlot 11.0 software (Systat Software, Inc., San Jose California USA). Differences between all the experimental groups were analyzed by ANOVA and intergroup comparisons were made using the Holm-Sidak test. For parameters not having a normal distribution, the Kruskal-Wallis test followed by Dunn's post-hoc test was used. The level of p ≤ 0.05 was considered statistically significant.

## Supporting information

S1 Fig Parasitemia analysis. **A**) Parasitemia (%) in ECM and NoECM rats *versus* time in days after infection. **B)** Parasitemia (Log%+1) evolution in ECM and NoECM in function of days aligned on baseline parasitemia at 5%. **C)** log (Parasitemia + 1) evolution in Sprague Dawley rats infected by K173 with ECM (n = 3) and NoECM (n = 3), which survived after a total parasitic clearance. All data are represented by the mean ± standard error of mean (SEM).
(TIF)

S2 Fig Discriminant analysis hematology. PLS-DA: Partial Least Square Discriminant Analysis is a supervised method designed to classify samples (here the Y class vector is ECM/NoECM) and identified the most predictive variables in the X-matrix of predictors. On actual values aligned DAY 2, performance of the fitting (“Leave-one-out” cross-validation method) gives an error rate of 28%. PLS-DA Hemato (CTRL excluded): error rate 28%. The distance between labels represents the correlation of parameters. The parameters are correlated with the closest class (ECM or NoECM). For example, high values for LY globally correspond to NoECM and low for ECM, while high values of RBC and superimposed parameters on the graph are associated with ECM. Red blood cell (RBC), white blood cell (WBC), platelet (Plt), granulocytes (GR), lymphocytes (LY), thrombocrite (THT), monocytes (MO), mean cell volume (MCV), mean cell hemoglobin (MCH), mean corpuscular hemoglobin concentration (MCHC), red blood cell distribution width (RDW), mean platelet volume (MPV).
(TIF)

S3 Fig **Mean Corpuscular Hemoglobin (MCH) (A) and Red Cell Distribution Width (RDW) (B)** during the course of K173 infection in ECM (n = 17), NoECM (n = 22) and Control (CTRL) (n = 13) groups. The hematological parameters are aligned from and on the basis of day D when parasitemia was estimated at 5% (S1B Fig). All data are represented by the mean ± standard error of mean (SEM).
(TIF)

S4 Fig Evolution of Platelet Distribution Width (PDW) during the course of K173 infection in ECM (n = 17), NoECM (n = 22) and Control (CTRL) (n = 13) groups. The hematological parameters are aligned from and on the basis of the day D when parasitemia was estimated at 5% (S1B Fig). All data are represented by the mean ± standard error of mean (SEM).
(TIFF)

S5 Fig **Counts of granulocytes (A), monocytes (B) and lymphocytes (C)** during the course of K173 infection in ECM (n = 17), NoECM (n = 22) and Control (CTRL) (n = 13) groups. Parameters are aligned from and on the basis of day D when parasitemia was estimated at 5% (S1B Fig). All data are represented by the mean ± standard error of mean (SEM).
(TIF)

S6 Fig Discriminant analysis of cytokines (Delta CT) on Delta CT values. PLS-DA: Partial Least Square Discriminant Analysis is a supervised method designed to classify samples (here the Y class vector is ECM/NoECM) and identify the most predictive variables in the X-matrix of predictors. Performance of the fitting (“Leave-one-out” cross-validation method) gives an error rate of 13%. The distance between labels represents the correlation of parameters. The parameters are correlated with the closest class (ECM or NoECM) in the graph. For example, high values for IL10 and IL6 were globally high for ECM and low for NoECM rats. Other parameters, close to the centre or in the northwest quarter are globally not really informative for the discrimination as they are equidistant from ECM and NoECM.
(TIF)

S1 Table Hematological values of SD rats infected by K173 after the parasitemia reached 5% (Day D). Pairwise comparisons were performed with Tukey adjustment post-analysis of the covariance with repeated measurements on days D+1, D+2, D+3.
(PDF)

S2 Table Biochemical parameters of infected (ECM, NoECM) and control (CTRL) SD rats. NoECM rats were divided in 2 groups: NoECM_LP_ with lower parasitemia (mean parasitemia = 26.5%) close to the parasitemia of ECM rats (mean parasitemia = 21.8%), and NoECM_HP_ with hyperparasitemia (mean parasitemia = 61.4%).
(TIF)

S3 Table Cytokine expression analysis in brains from ECM, NoECM and control rats (global ANOVA is followed by post hoc test for pairwise comparisons with Tukey adjustment). (TIF)

S4 Table List of genes and primer sequences used in RT-qPCR assays for cerebral cytokine analysis. (TIF)

S5 Table Histological features of K173 malaria infected SD rats and Control rats in no brain lavage (NBL). (TIF)

S6 Table Histological features of K173 malaria infected SD rats and Control rats after brain lavage (BL). (TIF)

S1 Text Statistical analysis: Detail of tests used. (PDF)
