# Supplementary figures and images for: Young Sprague Dawley rats infected by Plasmodium berghei: A relevant experimental model to study cerebral malaria
Source: PLoS One. 2017 Jul 24;12(7):e0181300. doi: 10.1371/journal.pone.0181300 (PMC5524346; doi:10.1371/journal.pone.0181300)

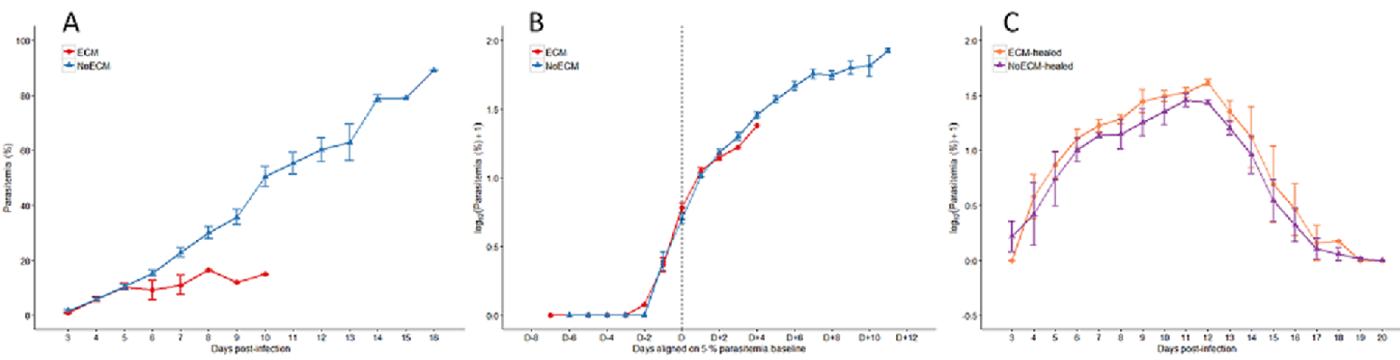

Supplement: S1 Fig — A) Parasitemia (%) in ECM and NoECM rats versus time in days after infection. B) Parasitemia (Log%+1) evolution in ECM and NoECM in function of days aligned on baseline parasitemia at 5%. C) log (Parasitemia + 1) evolution in Sprague Dawley rats infected by K173 with ECM (n = 3) and NoECM (n = 3), which survived after a total parasitic clearance. All data are represented by the mean ± standard error of mean (SEM). (TIF) [file pone.0181300.s001.tif]

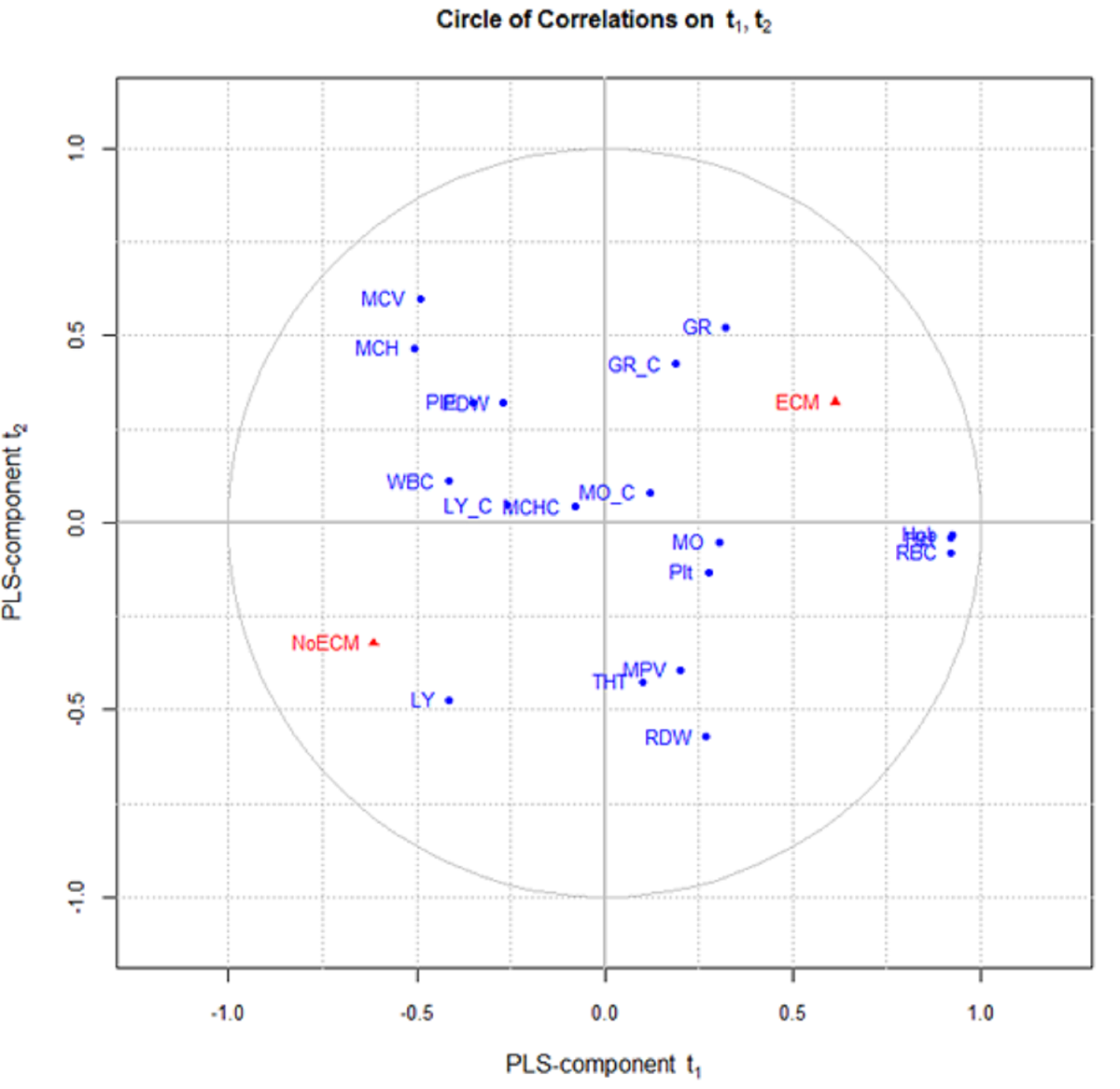

Supplement: S2 Fig — PLS-DA: Partial Least Square Discriminant Analysis is a supervised method designed to classify samples (here the Y class vector is ECM/NoECM) and identified the most predictive variables in the X-matrix of predictors. On actual values aligned DAY 2, performance of the fitting (“Leave-one-out” cross-validation method) gives an error rate of 28%. PLS-DA Hemato (CTRL excluded): error rate 28%. The distance between labels represents the correlation of parameters. The parameters are correlated with the closest class (ECM or NoECM). For example, high values for LY globally correspond to NoECM and low for ECM, while high values of RBC and superimposed parameters on the graph are associated with ECM. Red blood cell (RBC), white blood cell (WBC), platelet (Plt), granulocytes (GR), lymphocytes (LY), thrombocrite (THT), monocytes (MO), mean cell volume (MCV), mean cell hemoglobin (MCH), mean corpuscular hemoglobin concentration (MCHC), red blood cell distribution width (RDW), mean platelet volume (MPV). (TIF) [file pone.0181300.s002.tif]

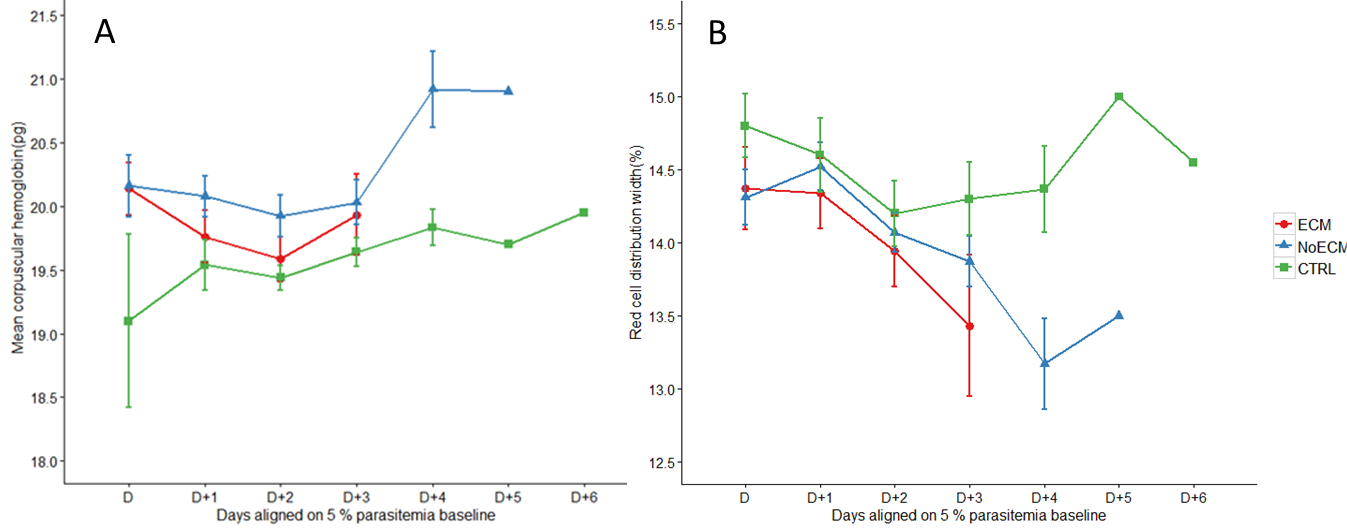

Supplement: S3 Fig — Mean Corpuscular Hemoglobin (MCH) (A) and Red Cell Distribution Width (RDW) (B) during the course of K173 infection in ECM (n = 17), NoECM (n = 22) and Control (CTRL) (n = 13) groups. The hematological parameters are aligned from and on the basis of day D when parasitemia was estimated at 5% (S1B Fig). All data are represented by the mean ± standard error of mean (SEM). (TIF) [file pone.0181300.s003.tif]

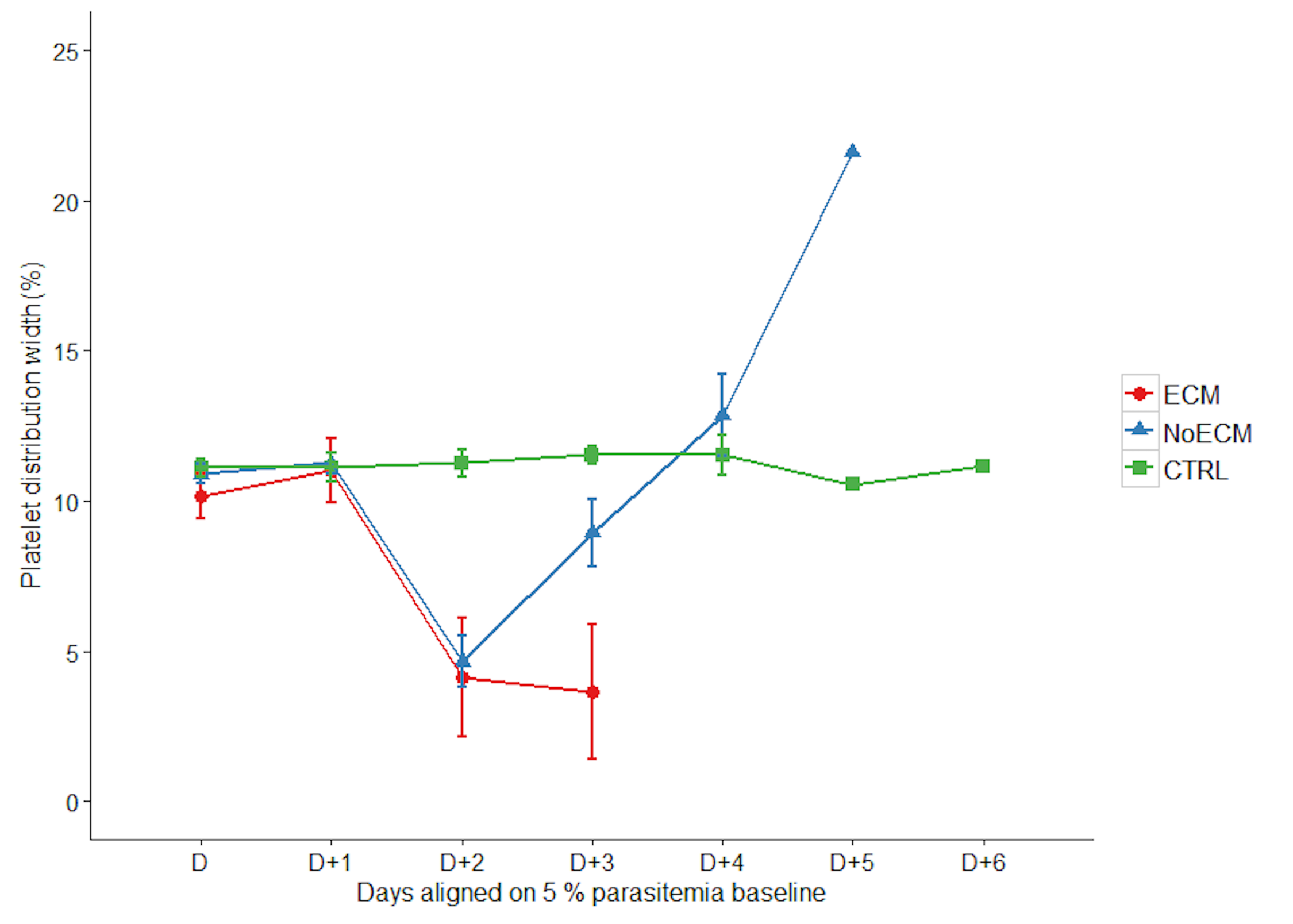

Supplement: S4 Fig — The hematological parameters are aligned from and on the basis of the day D when parasitemia was estimated at 5% (S1B Fig). All data are represented by the mean ± standard error of mean (SEM). (TIFF) [file pone.0181300.s004.tiff]

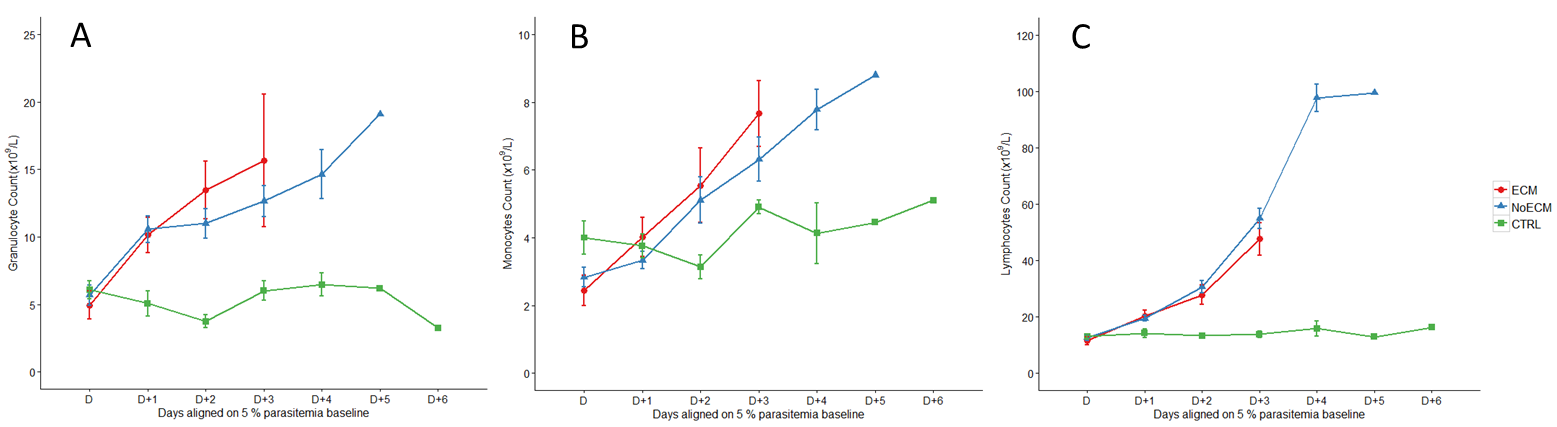

Supplement: S5 Fig — Counts of granulocytes (A), monocytes (B) and lymphocytes (C) during the course of K173 infection in ECM (n = 17), NoECM (n = 22) and Control (CTRL) (n = 13) groups. Parameters are aligned from and on the basis of day D when parasitemia was estimated at 5% (S1B Fig). All data are represented by the mean ± standard error of mean (SEM). (TIF) [file pone.0181300.s005.tif]

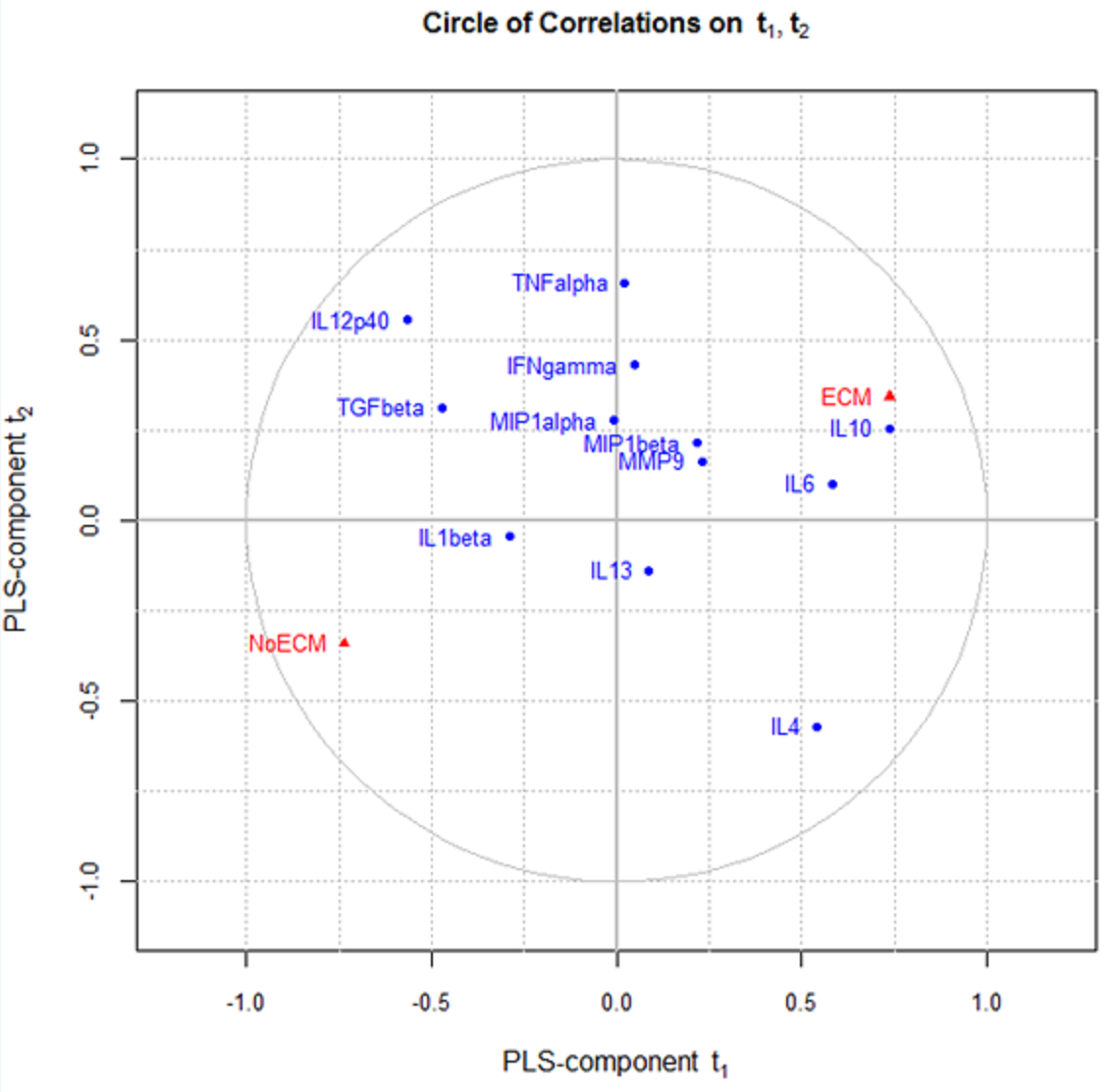

Supplement: S6 Fig — PLS-DA: Partial Least Square Discriminant Analysis is a supervised method designed to classify samples (here the Y class vector is ECM/NoECM) and identify the most predictive variables in the X-matrix of predictors. Performance of the fitting (“Leave-one-out” cross-validation method) gives an error rate of 13%. The distance between labels represents the correlation of parameters. The parameters are correlated with the closest class (ECM or NoECM) in the graph. For example, high values for IL10 and IL6 were globally high for ECM and low for NoECM rats. Other parameters, close to the centre or in the northwest quarter are globally not really informative for the discrimination as they are equidistant from ECM and NoECM. (TIF) [file pone.0181300.s006.tif]

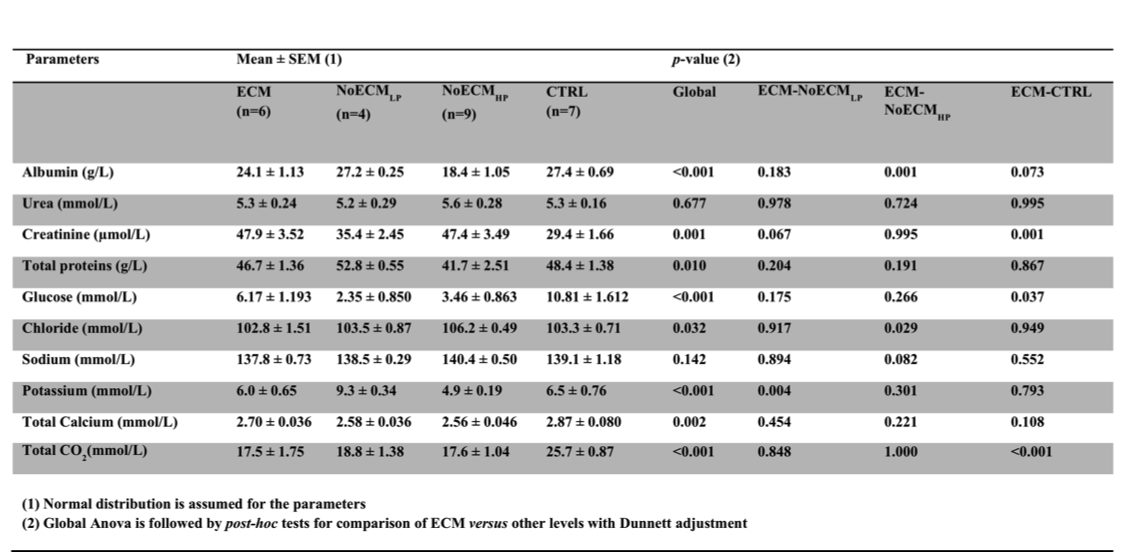

Supplement: S2 Table — NoECM rats were divided in 2 groups: NoECMLP with lower parasitemia (mean parasitemia = 26.5%) close to the parasitemia of ECM rats (mean parasitemia = 21.8%), and NoECMHP with hyperparasitemia (mean parasitemia = 61.4%). (TIF) [file pone.0181300.s008.tif]

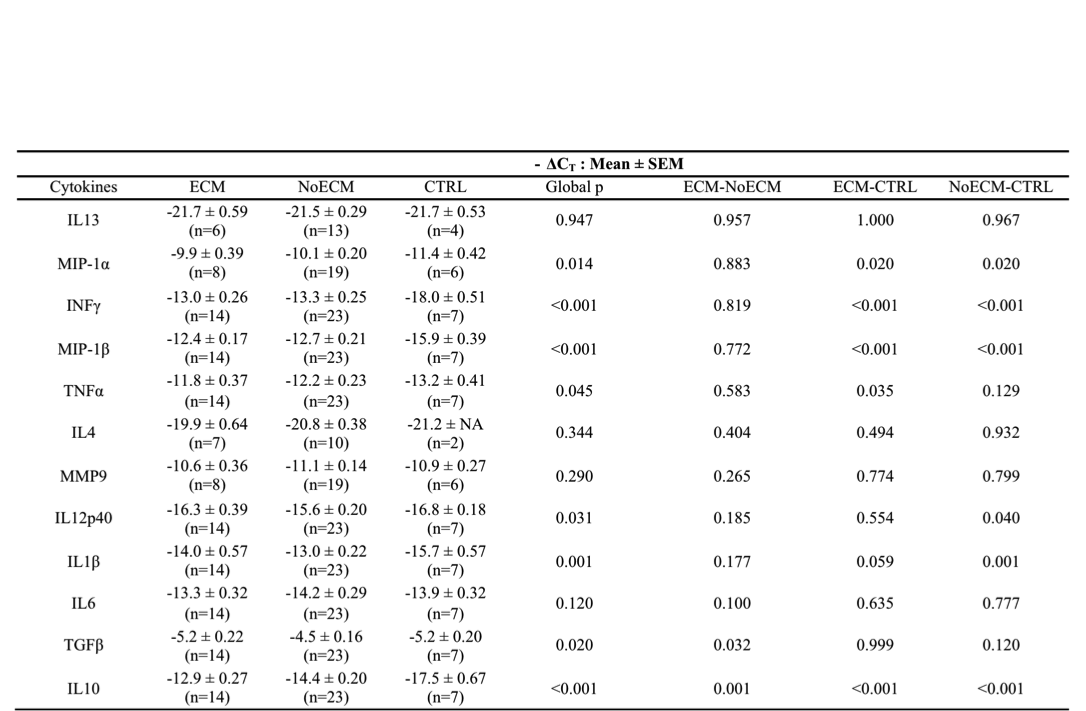

Supplement: S3 Table — (TIF) [file pone.0181300.s009.tif]

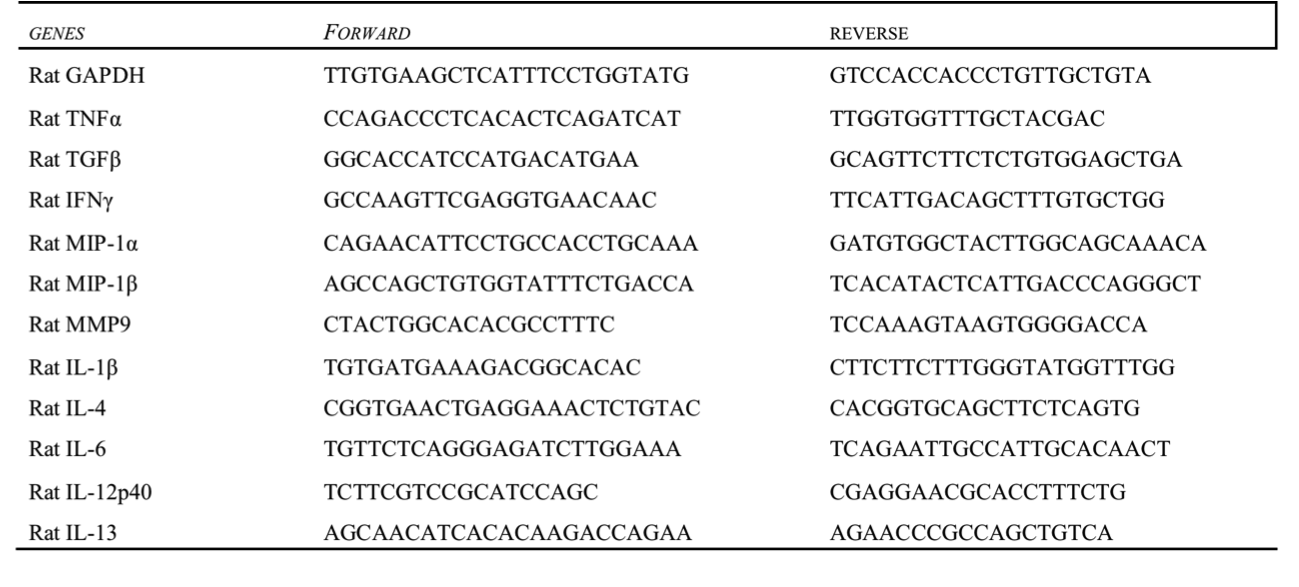

Supplement: S4 Table — (TIF) [file pone.0181300.s010.tif]

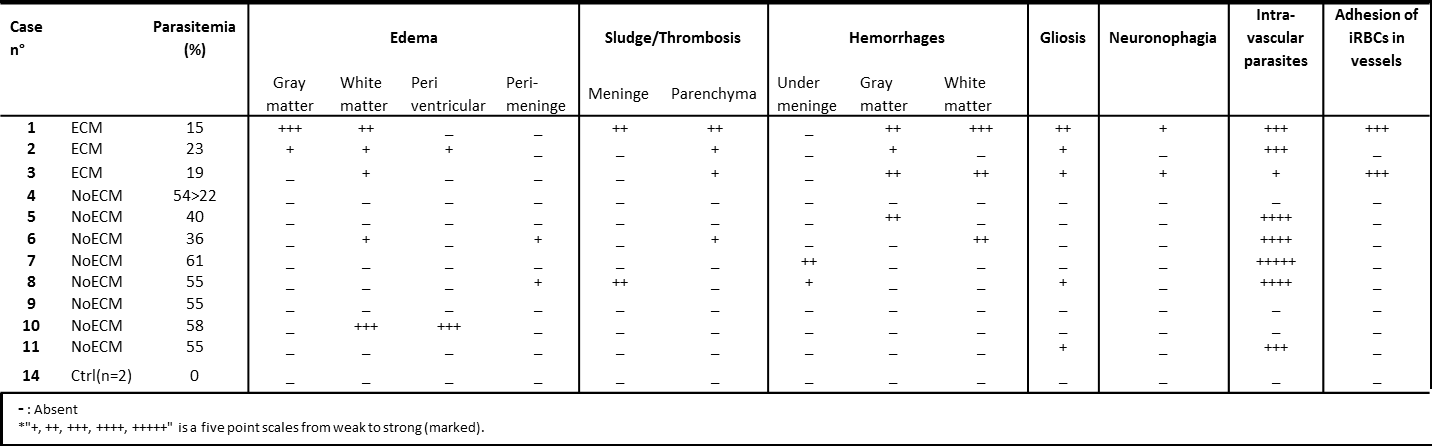

Supplement: S5 Table — (TIF) [file pone.0181300.s011.tif]

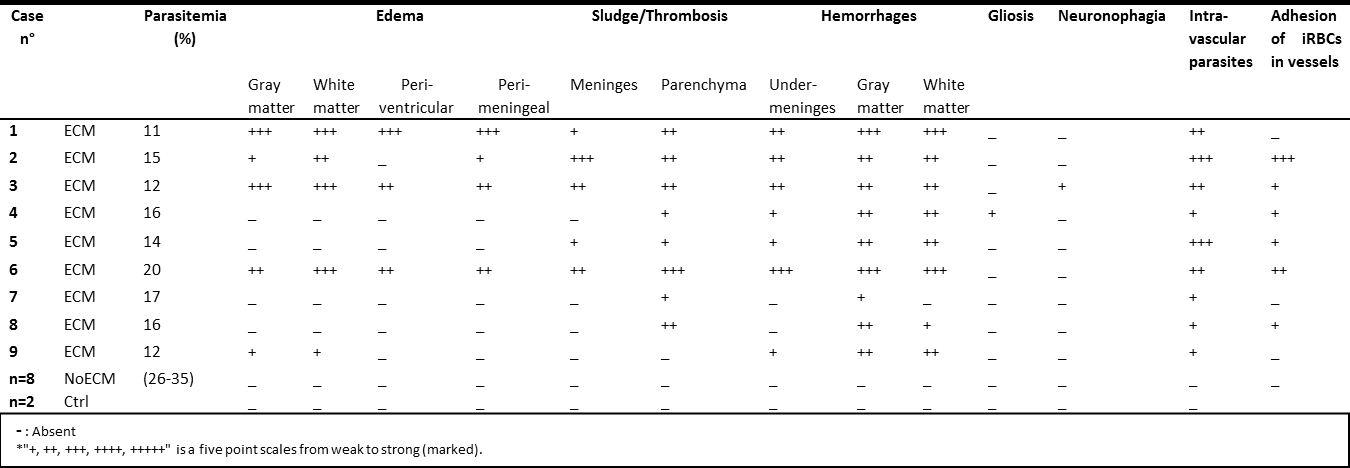

Supplement: S6 Table — (TIF) [file pone.0181300.s012.tif]
