# Supplementary material for: Young Sprague Dawley rats infected by Plasmodium berghei: A relevant experimental model to study cerebral malaria
Source: PLoS One. 2017 Jul 24;12(7):e0181300. doi: 10.1371/journal.pone.0181300 (PMC5524346; doi:10.1371/journal.pone.0181300)
Supplement: S1 Table — Pairwise comparisons were performed with Tukey adjustment post-analysis of the covariance with repeated measurements on days D+1, D+2, D+3. (PDF) [file pone.0181300.s007.pdf]

Parameters	Days	ECM	NoECM	CTRL	ECM - NoECM	ECM - CTRL	NoECM - CTRL
White Blood Cell (WBC) count (x10^9*/L)	D+1	30.8 ± 3.15 (n=7)	36.5 ± 5.93 (n=6)	19.1 ± 0.35 (n=3)	0.975	0.366	0.302
	D+2	48.4 ± 5.98 (n=11)	46.9 ± 6.05 (n=8)	18.4 ± 1.23 (n=3)	0.793	0.001	0.007
	D+3	NA	81.3 ± 18.32 (n=5)	14.4 ± NA (n=1)	NA	NA	<0.001
% Lymphocytes in WBC (%)	D+1	72.1 ± 1.59 (n=7)	71.1 ± 2.47 (n=5)	82.0 ± 1.02 (n=3)	0.968	0.526	0.661
	D+2	66.3 ± 1.97 (n=11)	74.3 ± 2.24 (n=8)	77.2 ± 1.11 (n=3)	0.004	0.215	0.999
	D+3	NA	78.6 ± 3.24 (n=5)	84.7 ± NA (n=1)	NA	NA	0.998
% Monocytes in WBC (%)	D+1	13.7 ± 1.34 (n=7)	14.6 ± 0.81 (n=5)	12.4 ± 0.72 (n=3)	0.912	0.997	0.951
	D+2	16.3 ± 0.66 (n=11)	13.0 ± 0.84 (n=8)	14.7 ± 0.35 (n=3)	0.003	0.905	0.461
	D+3	NA	11.0 ± 1.24 (n=5)	10.1 ± NA (n=1)	NA	NA	0.995
% Granulocytes in WBC (%)	D+1	14.2 ± 1.15 (n=7)	14.3 ± 1.99 (n=5)	5.7 ± 0.48 (n=3)	0.920	0.366	0.536
	D+2	17.3 ± 1.67 (n=11)	12.7 ± 1.93 (n=8)	8.1 ± 1.23 (n=3)	0.073	0.140	0.710
	D+3	NA	10.4 ± 2.32 (n=5)	5.2 ± NA (n=1)	NA	NA	0.964
Lymphocyte Count (x10^9*/L)	D+1	22.2 ± 2.48 (n=7)	27.7 ± 3.88 (n=5)	15.6 ± 0.38 (n=3)	0.983	0.539	0.607
	D+2	31.6 ± 4.03 (n=11)	35.3 ± 5.36 (n=8)	14.1 ± 1.08 (n=3)	0.922	0.081	0.055
	D+3	NA	66.2 ± 16.79 (n=5)	12.2 ± NA (n=1)	NA	NA	<0.001
Monocyte Count (x10^9*/L)	D+1	3.9 ± 0.31 (n=7)	5.8 ± 1.11 (n=5)	2.3 ± 0.12 (n=3)	0.537	0.747	0.377
	D+2	8.0 ± 1.01 (n=11)	5.7 ± 0.51 (n=8)	2.7 ± 0.12 (n=3)	0.051	0.017	0.265
	D+3	NA	8.2 ± 1.44 (n=5)	1.4 ± NA (n=1)	NA	NA	0.001
Granulocyte Count (x10^9*/L)	D+1	4.6 ± 0.68 (n=7)	6.0 ± 1.58 (n=5)	1.2 ± 0.12 (n=3)	0.988	0.640	0.613
	D+2	8.8 ± 1.39 (n=11)	5.8 ± 0.92 (n=8)	1.6 ± 0.20 (n=3)	0.035	0.029	0.411
	D+3	NA	7.1 ± 0.53 (n=5)	0.8 ± NA (n=1)	NA	NA	0.159

Parameters	Days	ECM	NoECM	CTRL	ECM - NoECM	ECM - CTRL	NoECM - CTRL
Red Blood Cell Count (x10^12*/L)	D+1	6.0 ± 0.18 (n=7)	5.6 ± 0.35 (n=6)	5.9 ± 0.13 (n=3)	0.977	0.895	0.961
	D+2	6.4 ± 0.39 (n=11)	5.0 ± 0.36 (n=8)	6.3 ± 0.13 (n=3)	0.036	0.935	0.407
	D+3	NA	5.0 ± 0.34 (n=6)	5.8 ± NA (n=1)	NA	NA	0.845
Hemoglobin (g/dL)	D+1	11.1 ± 0.24 (n=7)	10.3 ± 0.63 (n=6)	11.0 ± 0.15 (n=3)	0.773	0.955	0.975
	D+2	11.7 ± 0.72 (n=11)	9.1 ± 0.65 (n=8)	11.1 ± 0.13 (n=3)	0.005	0.744	0.386
	D+3	NA	9.1 ± 0.67 (n=6)	11.0 ± NA (n=1)	NA	NA	0.649
Hematocrit (%)	D+1	40.1 ± 0.95 (n=7)	36.5 ± 2.01 (n=6)	37.5 ± 0.55 (n=3)	0.713	0.863	0.998
	D+2	42.0 ± 2.67 (n=11)	32.9 ± 2.67 (n=8)	40.5 ± 0.38 (n=3)	0.026	0.923	0.385
	D+3	NA	35.5 ± 3.08 (n=6)	37.0 ± NA (n=1)	NA	NA	0.976
Mean corpuscular volume (fL)	D+1	66.4 ± 0.78 (n=7)	65.2 ± 0.75 (n=6)	64.0 ± 0.58 (n=3)	0.363	0.897	0.881
	D+2	65.9 ± 0.71 (n=11)	66.1 ± 1.24 (n=8)	64.3 ± 0.88 (n=3)	0.830	0.977	0.986
	D+3	NA	70.8 ± 1.97 (n=6)	64.0 ± NA (n=1)	NA	NA	0.015
Mean corpuscular hemoglobin (pg)	D+1	18.4 ± 0.19 (n=7)	18.3 ± 0.11 (n=6)	18.8 ± 0.18 (n=3)	0.995	0.472	0.534
	D+2	18.4 ± 0.20 (n=11)	18.3 ± 0.10 (n=8)	17.8 ± 0.22 (n=3)	0.854	0.289	0.524
	D+3	NA	18.3 ± 0.23 (n=6)	18.9 ± NA (n=1)	NA	NA	0.303
Mean corpuscular hemoglobin concentration (g/dL)	D+1	27.7 ± 0.10 (n=7)	28.1 ± 0.31 (n=6)	29.4 ± 0.22 (n=3)	0.310	0.251	0.859
	D+2	28.0 ± 0.21 (n=11)	27.8 ± 0.52 (n=8)	27.5 ± 0.18 (n=3)	0.604	0.669	0.328
	D+3	NA	25.9 ± 0.61 (n=6)	29.8 ± NA (n=1)	NA	NA	0.001
Red cell distribution width (%)	D+1	14.4 ± 0.15 (n=7)	14.2 ± 0.38 (n=6)	14.6 ± 0.15 (n=3)	0.944	0.490	0.641
	D+2	14.5 ± 0.21 (n=11)	13.9 ± 0.15 (n=8)	14.8 ± 0.47 (n=3)	0.367	0.412	0.096
	D+3	NA	13.7 ± 0.42 (n=6)	14.6 ± NA (n=1)	NA	NA	0.231

Parameters	Days	ECM	NoECM	CTRL	ECM - NoECM	ECM - CTRL	NoECM - CTRL
Platelet Count (x10^9*/L)	D+1	233.0 ± 41.59 (n=6)	245.8 ± 70.25 (n=4)	657.5 ± NA (n=2)	0.765	<0.001	<0.001
	D+2	78.9 ± 14.96 (n=11)	176.8 ± 87.42 (n=6)	900.3 ± 110.11 (n=3)	0.496	<0.001	<0.001
	D+3	NA	129.2 ± 32.04 (n=6)	NA	NA	NA	NA
Mean platelet volume (fL)	D+1	7.4 ± 0.20 (n=7)	7.6 ± 0.58 (n=6)	6.7 ± 0.47 (n=3)	0.677	0.267	0.672
	D+2	7.5 ± 0.26 (n=11)	7.7 ± 0.34 (n=8)	6.4 ± 0.24 (n=3)	0.668	0.122	0.427
	D+3	NA	8.7 ± 0.36 (n=6)	6.9 ± NA (n=1)	NA	NA	0.507
Platelet distribution width (%)	D+1	10.5 ± 0.63 (n=7)	9.3 ± 1.81 (n=4)	10.9 ± 0.38 (n=3)	0.798	0.983	0.805
	D+2	4.2 ± 1.00 (n=11)	9.5 ± 1.40 (n=6)	11.4 ± 0.20 (n=3)	0.004	0.011	0.797
	D+3	NA	10.3 ± 2.01 (n=5)	5.1 ± NA (n=1)	NA	NA	0.309
Thrombocrit (%)	D+1	0.20 ± 0.037 (n=7)	0.17 ± 0.046 (n=4)	0.40 ± 0.060 (n=3)	0.879	0.019	0.015
	D+2	0.06 ± 0.012 (n=11)	0.13 ± 0.059 (n=6)	0.58 ± 0.079 (n=3)	0.600	<0.001	<0.001
	D+3	NA	0.11 ± 0.022 (n=6)	0.05 ± NA (n=1)	NA	NA	0.647
