# Supplementary material for: Young Sprague Dawley rats infected by Plasmodium berghei: A relevant experimental model to study cerebral malaria
Source: PLoS One. 2017 Jul 24;12(7):e0181300. doi: 10.1371/journal.pone.0181300 (PMC5524346; doi:10.1371/journal.pone.0181300)
Supplement: S1 Text — (PDF) [file pone.0181300.s013.pdf]

Text S1: Statistical analysis: detail description of tests used

Software

Data analyses were performed using:

- "R: A language and environment for statistical computing" (R Core Team (2016) and SAS/STAT® software 9.4 (SAS Institute Inc.) for hematological parameters, brain cytokine gene expressions and biochemistry data
- SigmaPlot 11.0 software (Systat Software, Inc., San Jose California USA) for data of permeability test (Evans blue and edema tests).
- Prism version 5.00 (GraphPad Software, USA) for blood cytokines data analysis

Hematology

Follow-up of hematological parameters along the days of the experiment was performed with a 2-way analysis of covariance with repeated measurements on days and animal's baseline value as covariate. The analysis considers a spherical correlation structure between times (e.g. variance of each time is assumed equal, covariance between times is assumed equal). *Post-hoc* tests for pairwise comparison of groups (ECM, NoECM and CTRL) at each time point are performed with Tukey's multiplicity adjustment. Data are summarized with Mean and Standard error of the mean (Mean ±SEM) by group and time point.

For the parasitemia parameter, the Log-Normal distribution assumption is retained. Geometric mean and geometric standard deviation are used to summarize data. A 2-way analysis of variance with repeated measurements is also performed; post-hoc tests at each time point do not need multiplicity adjustment as only ECM and NoECM groups are compared, Control is obviously constant to zero for parasitemia and withdrawn from the statistical model.

Parasitemia is primarily analyzed along the days post-infection (Day 0 to Day 10), then the strategy is to align the time range on the alternative animal baseline which is the day achieving the parasitemia closer to 5% value. This approach is intended to correct for late parasitemia responders. In practice the parasitemia was measured from day 4 after infection and for early responders the first value measured can be over than 10%. To adjust the baseline day to the relevant start of parasitemia for the animal, missing values at day 1, 2 and 3 are estimated with a log-linear interpolation approach between day 0 (where parasitemia is 0) and day 4 (first experimental assessment). Here, we assume the log distribution of the parasitemia and use log (y+1) transformation for the interpolation.

Other hematological parameters were aligned on the 5% parasitemia baseline and analyzed on the 3 first days.

As complementary analysis, a Partial Least Square - Discriminant Analysis (PLS-DA) was produced to describe the correlation structure of parameters dependent on the ECM/NoECM outcome. This model was implemented on day 2 after timeframe alignment on 5% parasitemia.

Cerebral Cytokines

Cytokine values are corrected by internal control GAPDH: Cytokine value = 2^(mean Ct GADPH - mean Ct cytokine) = $2^{-\Delta C_T}$

The expression induced a Log-Normal distribution for the cytokine measures, log2 (Cytokine value) is used for statistical analyses (i.e.: statistics performed on the difference of CT value minus internal reference GAPDH). Cytokine expression is summarized with the geometric mean and geometric standard deviation.

A preliminary two-way analysis of variance with ECM, NoECM, Ctrl outcome groups and lavaged /not lavaged brain showed that the interaction is not significant. The differences between outcome groups do not depend on whether the brain is lavaged or not. Therefore, lavaged /not lavaged brain has been removed from the model and a one-way analysis of variance on was performed to compare groups ECM, NoECM and Ctrl. Tukey's adjustment for multiplicity was applied on pairwise comparisons post-Anova.

As complementary analysis, a Partial Least Square Discriminant Analysis (PLS-DA) was produced to describe the correlation structure of parameters dependent on the ECM/NoECM outcome.

Biochemistry

As the sample size per group is low, distribution of the responses cannot be evaluated and so, normal distribution is assumed. A one-way analysis of variance is carried out followed by post-hoc tests for comparison of ECM groups versus $NoECM_{HP}$ (high parasitemia), $NoECMl_{LP}$ (low parasitemia) and control. Dunnett's adjustment for multiplicity is applied on post-hoc comparisons.

Evans Blue and edema tests

The data collected were analyzed using SigmaPlot 11.0 software (Systat Software, Inc., San Jose California USA). Differences between all the experimental groups were analyzed by ANOVA and intergroup comparisons were made using the Holm-Sidak test. For parameters not having a normal distribution, the Kruskal-Wallis test followed by Dunn's post-hoc test was used. The level of $p \leq 0.05$ was considered statistically significant.
